# The Electrogenic Na^+^/K^+^ Pump Is a Key Determinant of Repolarization Abnormality Susceptibility in Human Ventricular Cardiomyocytes: A Population-Based Simulation Study

**DOI:** 10.3389/fphys.2017.00278

**Published:** 2017-05-05

**Authors:** Oliver J. Britton, Alfonso Bueno-Orovio, László Virág, András Varró, Blanca Rodriguez

**Affiliations:** ^1^Department of Computer Science, University of OxfordOxford, UK; ^2^Department of Pharmacology and Pharmacotherapy, Faculty of Medicine, University of SzegedSzeged, Hungary

**Keywords:** human, repolarization, cardiac electrophysiology modeling, variability, sodium-potassium pump, Na^+^/K^+^ pump

## Abstract

**Background:** Cellular repolarization abnormalities occur unpredictably due to disease and drug effects, and can occur even in cardiomyocytes that exhibit normal action potentials (AP) under control conditions. Variability in ion channel densities may explain differences in this susceptibility to repolarization abnormalities. Here, we quantify the importance of key ionic mechanisms determining repolarization abnormalities following ionic block in human cardiomyocytes yielding normal APs under control conditions.

**Methods and Results:** Sixty two AP recordings from non-diseased human heart preparations were used to construct a population of human ventricular models with normal APs and a wide range of ion channel densities. Multichannel ionic block was applied to investigate susceptibility to repolarization abnormalities. I_Kr_ block was necessary for the development of repolarization abnormalities. Models that developed repolarization abnormalities over the widest range of blocks possessed low Na^+^/K^+^ pump conductance below 50% of baseline, and I_CaL_ conductance above 70% of baseline. Furthermore, I_NaK_ made the second largest contribution to repolarizing current in control simulations and the largest contribution under 75% I_Kr_ block. Reversing intracellular Na^+^ overload caused by reduced I_NaK_ was not sufficient to prevent abnormalities in models with low Na^+^/K^+^ pump conductance, while returning Na^+^/K^+^ pump conductance to normal substantially reduced abnormality occurrence, indicating I_NaK_ is an important repolarization current.

**Conclusions:** I_NaK_ is an important determinant of repolarization abnormality susceptibility in human ventricular cardiomyocytes, through its contribution to repolarization current rather than homeostasis. While we found I_Kr_ block to be necessary for repolarization abnormalities to occur, I_NaK_ decrease, as in disease, may amplify the pro-arrhythmic risk of drug-induced I_Kr_ block in humans.

## Introduction

Recent evidence points toward possible large variations in ion channel densities in cardiac cells, that are modulated by environmental and intrinsic factors including: circadian rhythms (Jeyaraj et al., 2012); exposure to hormones (Odening and Koren, 2014); to drugs (Xiao et al., 2008); to sustained change in pacing rate (Qi et al., 2008); to arrhythmias (Nattel et al., 2007); and to disease (Nass et al., 2008; Michael et al., 2009). Regulation of mRNA expression (Nattel et al., 2010) and further regulation of mRNA by miRNA (Kim, 2013) are two mechanisms by which cardiomyocytes can remodel their complement of ion channels in response to dynamic changes in cellular external conditions. Therefore, cells have the ability to adapt to environmental factors by modulating their ion channel densities while still maintaining their physiological function, represented in cardiomyocytes by the action potential (AP).

Aggressive external stimuli such as drugs or disease can sometimes challenge the stable behavior of cardiomyocytes leading to unexpected and potentially lethal abnormalities, even in tissue exhibiting apparently healthy behavior under normal conditions. In the case of the heart, its normal function can be disrupted by the presence of repolarization abnormalities, including early afterdepolarizations (EADs) and repolarization failure, which are rare and unpredictable pro-arrhythmic side-effects of disease and drug application. A wide range of ionic mechanisms have been identified as contributors to repolarization abnormalities, mostly through animal studies. Ionic mechanisms implicated include reduced repolarization reserve (Roden, 1998); L-type Ca^2+^ current (I_CaL_) reactivation (January and Riddle, 1989); and intracellular Ca^2+^ overload, driven by either external changes, or by modulation of the Na^+^/Ca^2+^ exchanger (I_NCX_) or Na^+^/K^+^ pump (I_NaK_) currents (Weiss et al., 2010; Despa and Bers, 2013; Bueno-Orovio et al., 2014; Shattock et al., 2015). Whereas animal studies have shown the importance of these individual mechanisms as contributors to repolarization abnormality generation under specific conditions, a quantitative understanding of the interactions and relative importance of these mechanisms in human ventricular cardiomyocytes affected by ionic current block is still missing.

In this study, we aim to quantitatively investigate the mechanisms underlying repolarization abnormalities in human cardiomyocytes, with a wide range of ionic profiles to consider variability in ionic properties. We specifically focus on investigating human cardiomyocytes yielding a normal AP under control conditions, using a population of human ventricular cardiomyocyte models (Britton et al., 2013) calibrated with experimental electrophysiological recordings.

## Methods

### Population of human ventricular cell models

The O'Hara-Virag-Varro-Rudy (ORd) model (O'Hara et al., 2011) of the human ventricular cardiomyocyte was used as the baseline model for our investigations, as it is one of the most recent, widely used and extensively tested models of the human ventricular cardiomyocyte, and is particularly well-suited for studying human ventricular repolarization, as key currents involved in repolarization and EAD formation (including I_Kr_, I_Ks_, I_K1_, and I_CaL_) are parameterized using data exclusively from undiseased human ventricular cardiomyocytes.

We constructed a population of 10,000 human models, each based on the ORd formulation but with differences in the conductance values for 9 key sarcolemmal currents, based on our assumptions that ionic conductances are highly variable, due to both intracellular factors and responses to external stimuli. The conductances varied were those with the most influence on the human ventricular AP: I_Na_ (fast Na^+^ current); I_NaL_ (late Na^+^ current); I_CaL_ (L-type Ca^2+^ current); I_to_ (transient outward K^+^ current); I_Kr_ (rapid delayed rectifier K^+^ current); I_Ks_ (slow delayed rectifier K^+^ current); I_K1_ (inward rectifier K^+^ current); I_NCX_; (Na^+^/Ca^2+^ exchanger current) and I_NaK_ (Na^+^/K^+^ pump current). Latin hypercube sampling (McKay et al., 1979) was used to sample sets of conductances uniformly while co-varying all conductances.

As in Britton et al. (2013) we selected a wide sampling range of 0–2 times the baseline value of each conductance to allow models with a wide variety of underlying ionic current configurations, including both increased and reduced channel densities, to be evaluated and potentially accepted into the population if they passed the filtering process. This range is necessarily an assumption as this range cannot be measured *in vivo*, and voltage clamp measurements of ionic current conductances in isolated cells are affected by the isolation process (Borg and Terracio, 1990). Using a lower sampling limit of 0 also allows the possibility of models with very low, potentially pathological values for some conductances, providing the resulting model can still produce a normal control AP. This choice of limit is intentional, to allow the investigation of abnormal ionic profiles that produce normal APs in control conditions but may have increased susceptibility to repolarization abnormalities under channel block. All conductance values in this study are given as scaling factors of the baseline ORd model's conductances.

### Experimental filtering to select normal APs in control conditions

Experimental recordings of human AP were obtained as described in O'Hara et al. (2011) from human right ventricular trabeculae and papillary tissue preparations of <2 cm in diameter, dissected from non-failing human hearts, perfused and paced at 1 Hz. Small tissue preparations, rather than isolated cells, were used in this study to avoid the damage to ion channels caused by the isolation process. AP recordings were acquired from these preparations using conventional microelectrode techniques from 62 experiments performed using tissue preparations from 37 hearts (16 females, 21 males, mean age 43 ± 13 years). The recordings used in this study were obtained from a database of recordings from multiple previous studies, all conducted by the same laboratory, which we then further analyzed to calculate biomarker values and experimental ranges. Further details of the preparation process and recording equipment are available in Jost et al. (2005). The preparations used in this study were obtained from hearts donated for research in compliance with the Declaration of Helsinki and approved by the Scientific and Research Ethical Committee of the Medical Scientific Board of the Hungarian Ministry of Health (ETT-TUKEB), under ethical approval No 4991-0/2010-1018EKU (339/PI/010).

**Figure 1:**
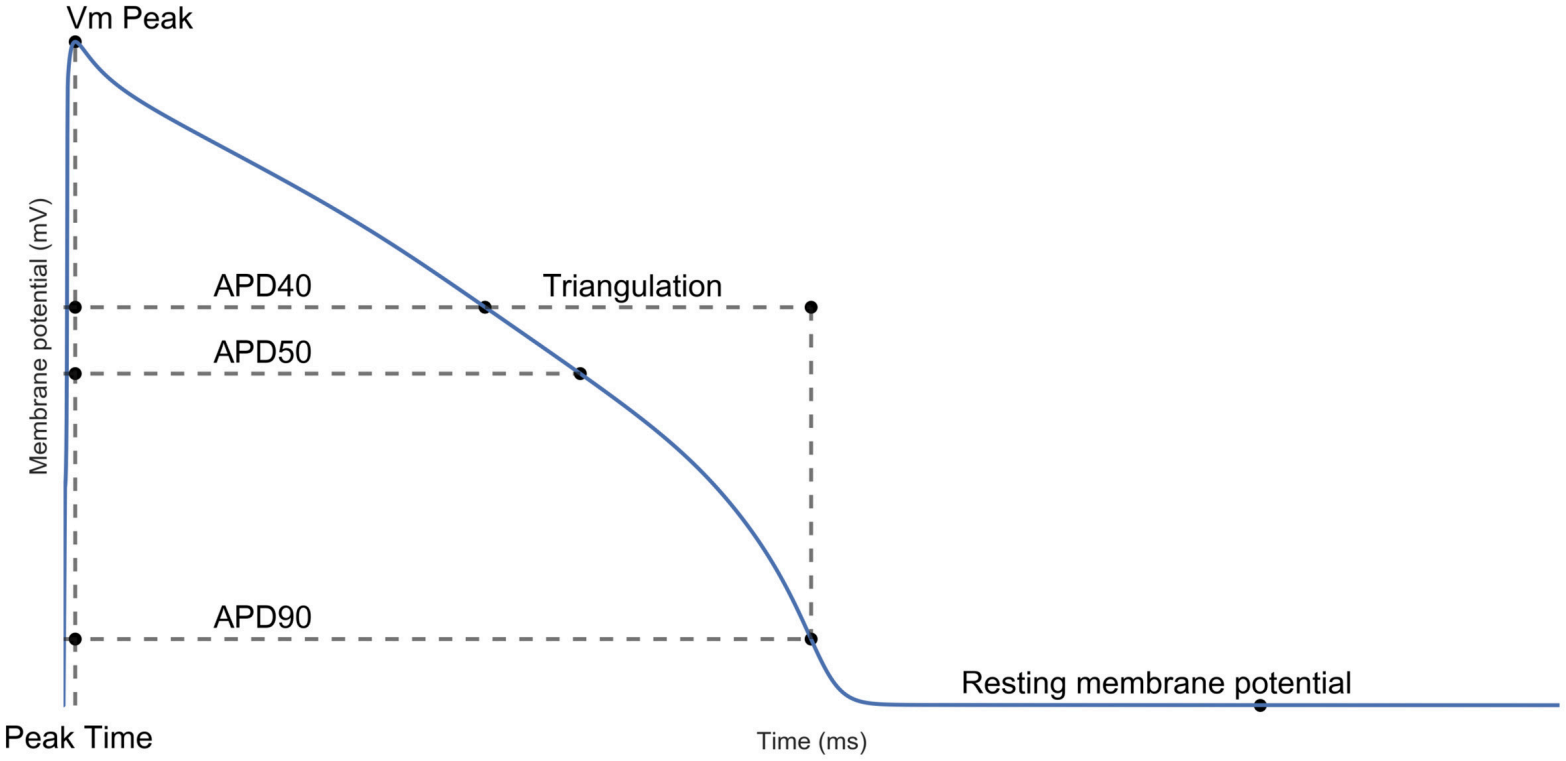
**Action potential biomarkers**. Peak membrane potential (V_m_ Peak); time of peak membrane potential (V_m_ Time); AP duration (APD) at 40/50/90% repolarization; triangulation and resting membrane potential (RMP). Each biomarker was calculated as defined in the Supplementary Material.

The data were analyzed to obtain the experimental ranges of seven biomarkers, which are shown in Figure 1. For each biomarker, experimental ranges were calculated from the minimum and maximum values observed in the data set at 1 Hz control conditions pacing, after excluding three clear outliers, one due to an exceptionally long APD90 (543 ms, data set mean = 293 ± 66 ms) and two due to exceptionally long times to peak (26 and 38 ms, data set mean = 6.7 ± 5.4 ms). The calculated biomarker ranges are displayed in Table S2. Biomarker values were generated for each experiment by calculating the value of each biomarker in the final recorded AP trace of the control portion of each experiment. These experimental ranges were then used to constrain the population of models to only those models that produced normal AP behavior under 1 Hz pacing in control conditions. We refrained from using any additional constraints on the population, to create a population of models with normal APs under normal pacing conditions, but with a wide range of underlying conductances, that we hypothesized would lead to a diverse range of susceptibilities to drug-induced repolarization abnormalities. Performing this filtering led to a total of 568 models of the initial 10,000 being accepted as having normal human ventricular APs; their parameter values are provided in Table S3 in the Supplementary Material.

### Detection and classification of repolarization abnormalities

Simulations were carried out with the 568 models to investigate the occurrence of repolarization abnormalities following block of I_Kr_, I_Ks_, I_K1_, and I_CaL_, often considered the key currents that contribute during repolarization (January and Riddle, 1989; Weiss et al., 2010; Varro and Baczko, 2011). We simulated block of all possible two-current pairs of these four currents (6 combinations) and for each pair simulated the 16 possible combinations of 4 block strengths (a 25, 50, 75, or 90% reduction in the appropriate conductance), for a total of 96 simulations performed on each of the 568 models. These simulations represent possible effects of non-selective blockers such as verapamil and dronedarone, both of which block I_Kr_ and I_CaL_ (Zhang et al., 1999; Varró et al., 2001), or combinations of selective blockers, e.g., dofetilide and HMR-1556 (Jost et al., 2013). We performed a systematic analysis of block levels, rather than matching specific compounds at set concentrations, to analyze the effects of a wide range of possible block scenarios, and because of the large uncertainties in IC50 values measured for concentration-dependent channel block (Polak et al., 2009).

**Figure 2:**
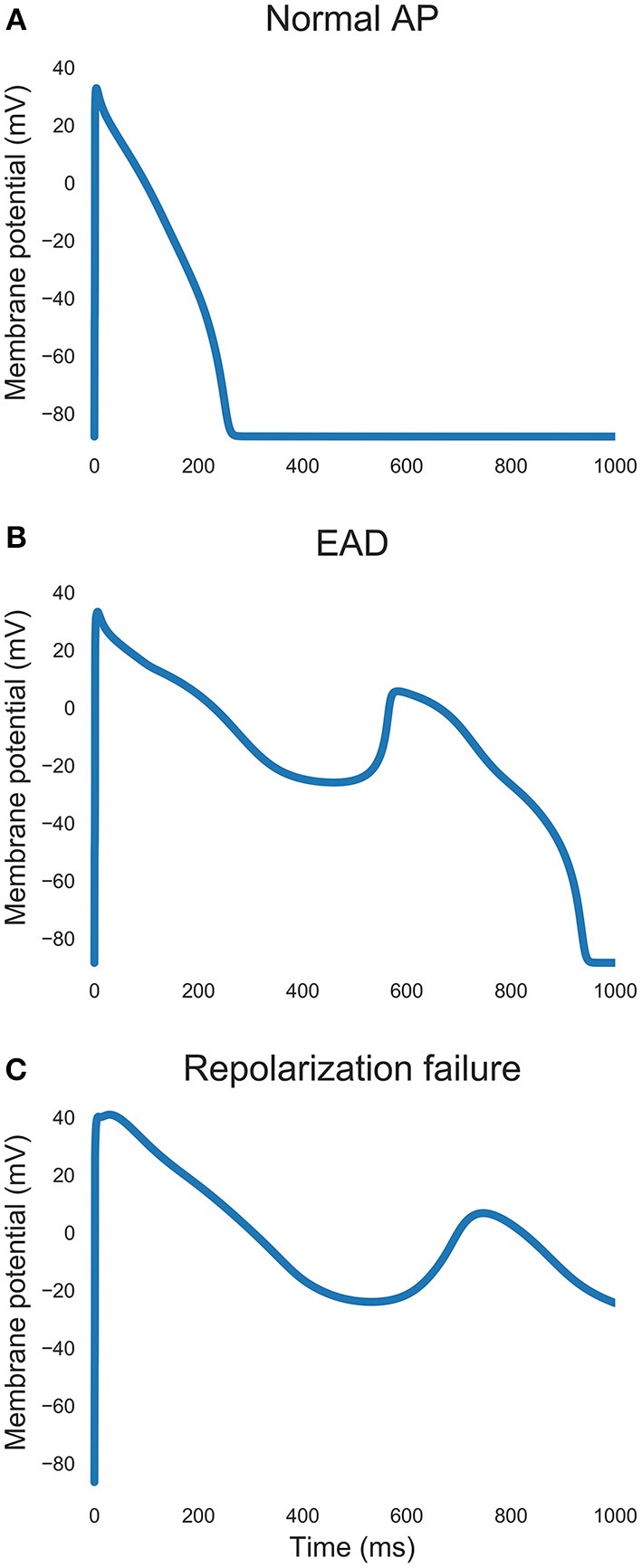
**Examples of action potential traces. (A)** Normal repolarization. **(B)** An early afterdepolarization. **(C)** Repolarization failure.

Repolarization abnormalities (Figure 2) were detected by analyzing the voltage-time gradient of the final two pacing cycles of a simulation. A positive gradient greater than 0.02 mV/ms more than 100 ms after the initial upstroke was used as an automatic detector of afterdepolarizations. We used a 100 ms delay to avoid models with either delayed initial upstroke or a spike and dome configuration from being misclassified as displaying afterdepolarizations.

For each drug block simulation we recorded which models developed repolarization abnormalities. Each model in the population was then categorized by the number of different channel block simulations in which they developed repolarization abnormalities. Models which did not develop repolarization abnormalities in any of the simulations were classified as not susceptible (NS), models which developed repolarization abnormalities in 1–9 of the simulations were classified as moderately susceptible (MS) and models which developed repolarization abnormalities in 10 or more of the simulations were classified as highly susceptible (HS). The threshold separating MS and HS models was selected as it marked a particularly large drop in the number of models displaying repolarization abnormalities in N or more simulations occurred between *N* = 8 and *N* = 10 (Figure S1).

### Statistical methods

We used the Mann-Whitney U-test to determine statistical differences between groups of biomarker values, as it is non-parametric and therefore did not require any assumption of the underlying distributions of biomarker values. Partial correlation coefficients were used to determine correlations between conductances, while controlling for the effects of all other co-varying conductances. Multiple comparisons were corrected for with Bonferroni correction.

A logistic regression model was developed to determine whether models that were highly susceptible to repolarization abnormalities could be classified based on their biomarker values alone. The z-scored values of the seven biomarkers used in this study, obtained from each model in the population, were used as features to train the logistic regression model, which was then used classify models in the test sets as either belonging to the HS category, or not. This classification was then compared to the true categorization. Ten-fold cross validation was used to generate multiple training and testing data sets.

Additional details on simulation protocols and the choice of model used in this study can be found in the Supplementary Material.

## Results

### Properties of the population of human ventricular cell models

**Figure 3:**
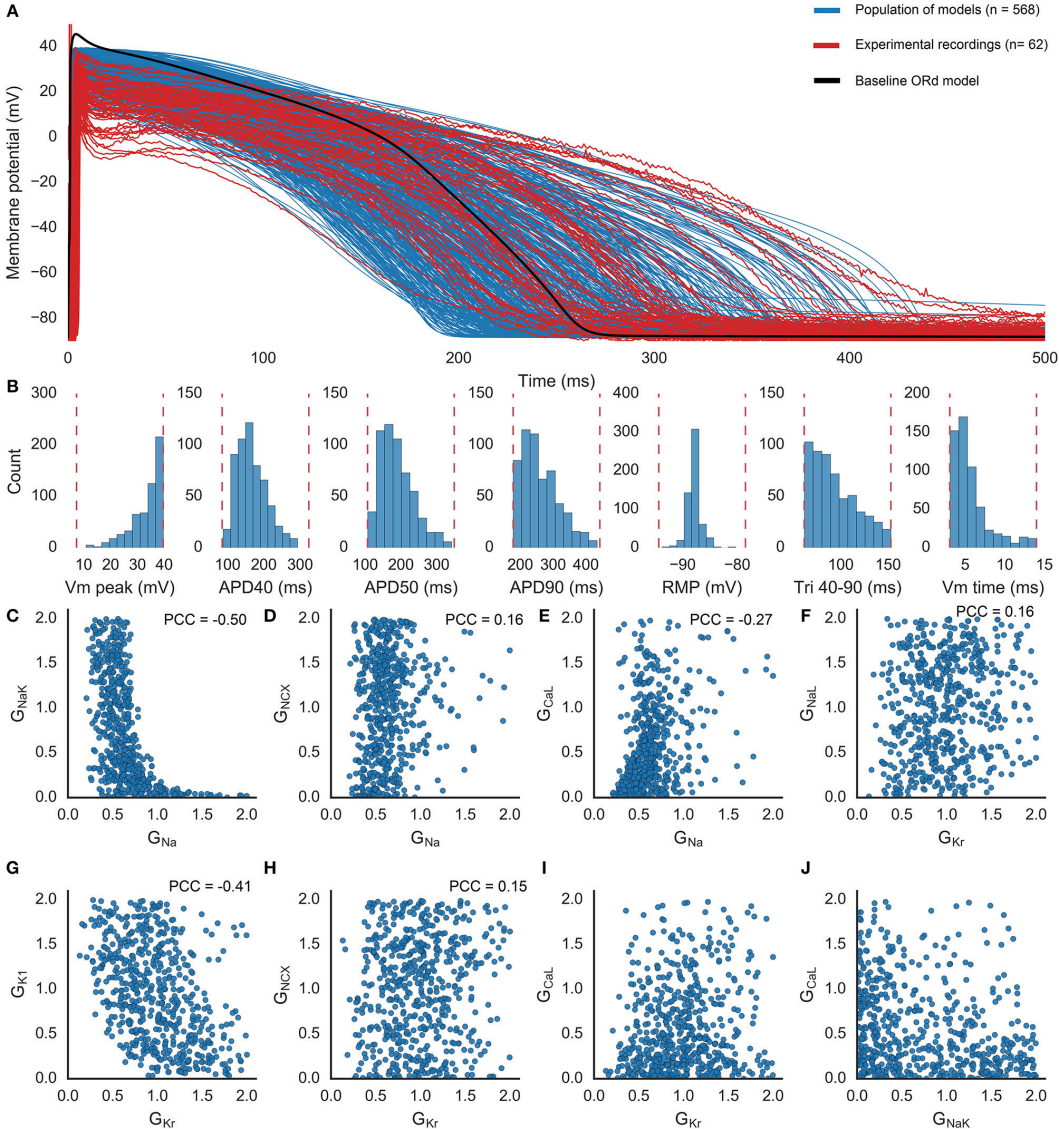
**Population of human ventricular cell models**. **(A)** APs obtained from control experimental recordings (red; *n* = 62 including three excluded outliers, two with long time to peak and one with long APD90); simulations using the models found to be within the experimental range (blue; *n* = 568); and the baseline ORd model (black), at 1 Hz pacing. **(B)** Distributions of each biomarker across the population of models. Dashed lines indicate the experimental range used to determine whether each model is accepted into the population. **(C–J)** Selected pairs of conductances across the population of models. Plots show scaling values relative to the value of each conductance in the baseline ORd model. Partial correlation coefficients (PCC) are shown for the six pairs of parameters with significant correlations (*p* < 0.05/36).

Figure 3A shows voltage traces from human models in the population spanning the range seen in experiments, and the baseline ORd model, which has a slightly higher peak membrane potential (45.6 mV) than the highest values seen in our data (39.6 mV), but otherwise would be a viable model in our population. Figure 3B shows that variability in conductances can reproduce the variability in the experimental biomarkers. One possible exception is RMP, which is highly dependent on the extracellular K^+^ concentration, considered constant in our simulations.

Figures 3C–J shows that most of the conductances span the full sampled range (0–2 times the ORd model's baseline value for that conductance), highlighting the robustness of the human AP against conductances variations. However, G_Na_, G_CaL_, and G_Kr_ are constrained by the calibration (Figures 3E,G). G_Na_ is confined to the smallest range, 92% of the models in the population have G_Na_ values clustered between 0.2 and 1. G_CaL_ values are densely distributed between 0 and 1 (81% of the population), so that few models have high G_CaL_ values (e.g., Figure 3E). Low values of G_Kr_ (below 0.13) are not present in the population (e.g., Figure 3G), suggesting that a minimum amount of I_Kr_ is necessary in the cell for successful repolarization under normal conditions, but the distribution of G_Kr_ values spans the rest of the sampled range. All possible pairings of ionic conductances within the population of models are shown in Figure S2. Results demonstrate that a normal AP can be generated by a wide variety of different balances of ionic currents, although these balances may have very different responses to changing conditions.

**Figure 4:**
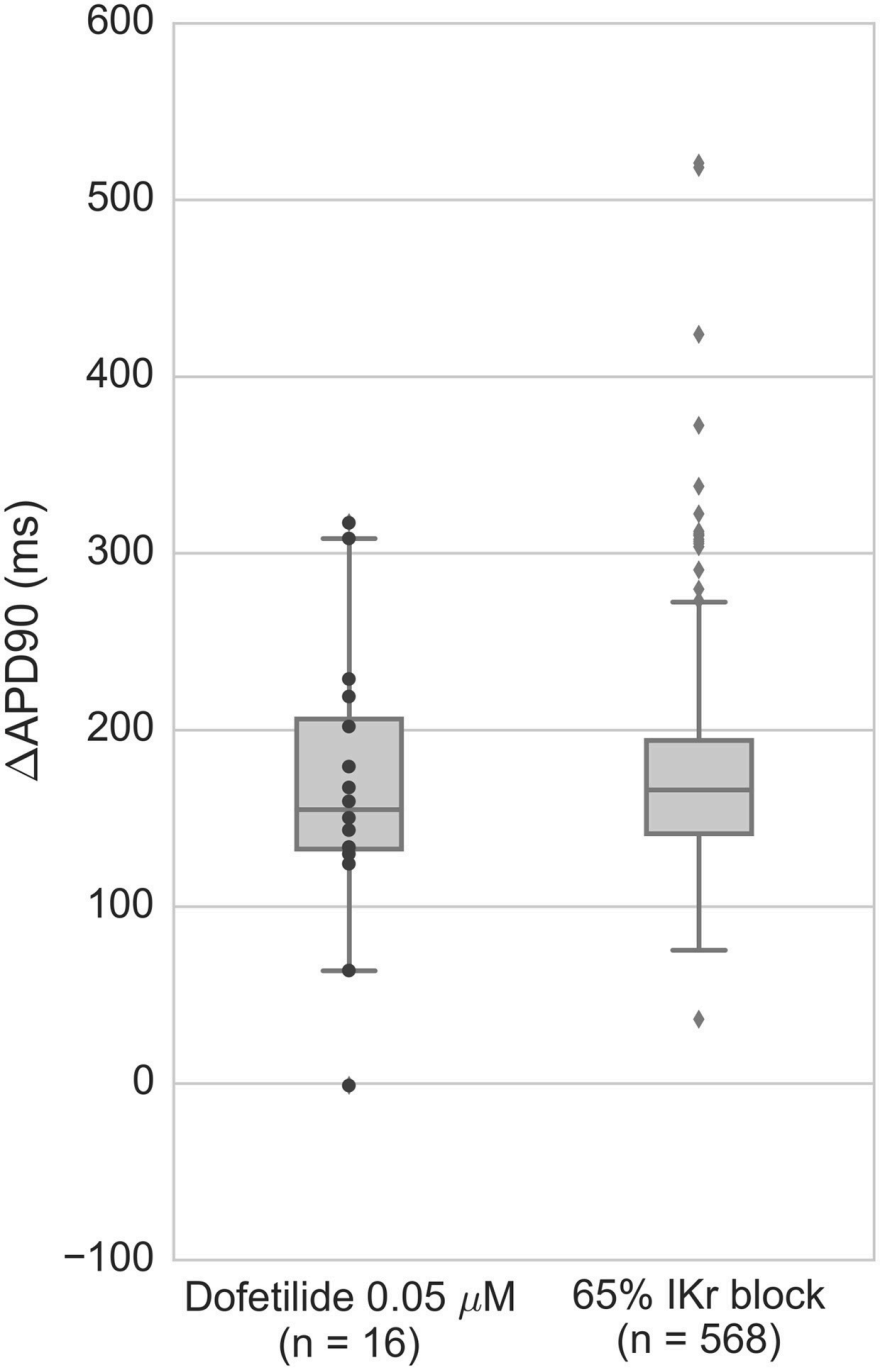
**APD prolongation of ventricular preparations under 0.05 μM dofetilide (***n*** = 16) compared to population of models under 65% I_**Kr**_ block, corresponding to the effects of 0.5 μM dofetilide using IC50 and Hill coefficient determined by Kramer et al. (**2013**)**. Models displaying repolarization abnormalities (7/568 models) were excluded from the figure. Black dots are experimental data points, gray dots are outliers (>1.5 times interquartile range from the nearest inner quartile).

To test whether the balance of repolarizing currents in the population of models resulted in a realistic range of APD prolongation values under I_Kr_ block, we compared the APD_90_ prolongation caused by application of 0.05 μM dofetilide (a selective I_Kr_ blocker) to the prolongation caused by equivalent I_Kr_ block on the population of models (Figure 4). Dofetilide was modeled as a single-pore I_Kr_ inhibitor with a Hill coefficient of 1.2 and an IC50 of 0.03 μM based on data from Kramer et al. (2013), giving a resulting I_Kr_ block of 65%. The distributions of APD prolongation overlap between experiment and the population. This provides confidence that a realistic balance of I_Kr_ and other repolarizing currents is captured by the population. There are some outliers in the population that are not seen in experiments, and 7/568 models were excluded from the figure due to developing repolarization abnormalities. This can be explained by the wider range of conductances explored in the population of models compared to the limited set of experimental data in non-diseased hearts.

### I_Kr_ block induces repolarization abnormalities, augmented by I_Ks_ and I_K1_ block, and opposed by I_CaL_ block

**Figure 5:**
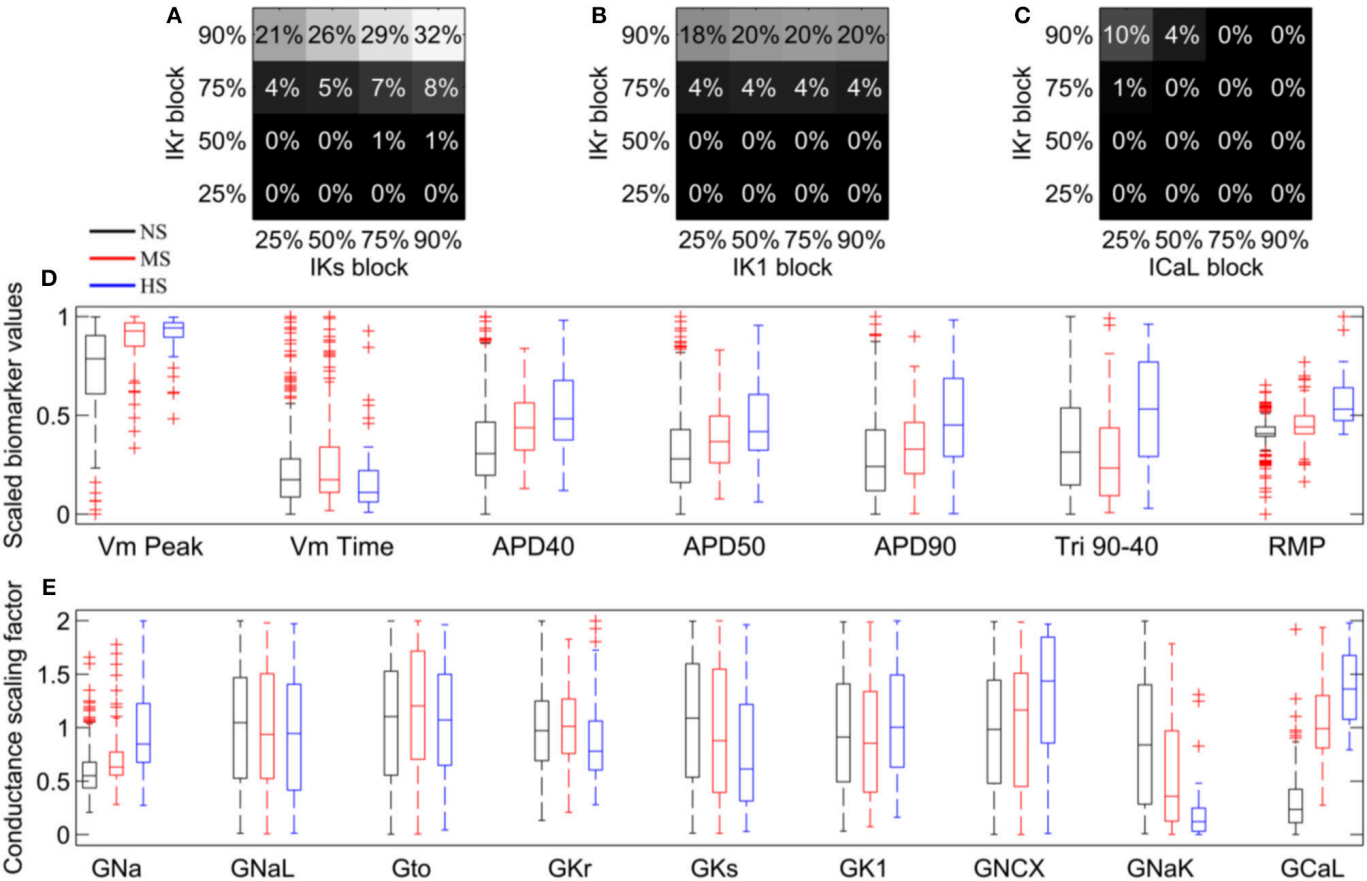
**Repolarization abnormality susceptibility across the population of models, in response to simulated drug block**. **(A–C)** Percentage of models in the population that displayed repolarization abnormalities in response to combinations of different strength blocks of I_Kr_, I_Ks_, I_K1_, and I_CaL_. No model developed repolarization abnormalities in any of the simulations in which I_Kr_ was not blocked, therefore these simulations are not included in the plots. **(D)** Biomarker distributions for NS (black), MS (red), and HS (blue) models in the population. **(E)** Conductance distributions for NS (black), MS (red), and HS (blue) models in the population.

To investigate our hypothesis that the different conductance profiles in our population would result in different levels of susceptibility to repolarization abnormalities, multiple channel block simulations blocking pairs of the currents I_Kr_, I_Ks_, I_K1_, and I_CaL_ at a range of block strengths, were conducted as described in Methods. The percentage of models in the population that generated abnormalities in each simulation, are shown in Figures 5A–C for the combinations of currents where abnormalities were detected in at least one simulation. Out of the 96 multichannel block simulations, 29 simulations caused at least one model to develop abnormalities. I_Kr_ block was the primary cause of repolarization abnormalities. No abnormalities were observed in any simulation where I_Kr_ was blocked by less than 50%, even though models with very low values of G_Kr_ were considered in the population. Block of either I_K1_ or I_Ks_ acted to augment I_Kr_ block and increased the number of models that produced abnormalities (Figures 5A,B), but were not sufficient to produce abnormalities without I_Kr_ block. I_CaL_ block attenuated the effects of I_Kr_ block, reducing the number of abnormalities seen in the population (Figure 5C). At 75% I_Kr_ block, 50% block of I_CaL_ was sufficient to abolish all repolarization abnormalities. For 90% I_Kr_ block, 75% I_CaL_ block was required to achieve the same effect. In both cases, even 25% block of I_CaL_ was sufficient to substantially reduce the incidence of repolarization abnormalities in the population, from 4 to 1% of models for 75% I_Kr_ block, and from 18 to 10% for 90% I_Kr_ block. Overall, when the population was exposed to both I_Kr_ and I_CaL_ block, few models produced abnormalities. However, we found that the APD prolongation caused by I_Kr_ block was only slightly reduced by I_CaL_ block (Figure S3).

### Low G_NaK_ and high G_CaL_ indicate particular susceptibility to repolarization abnormalities

To analyze how susceptibility to repolarization abnormalities depended on underlying conductance and biomarker values, we classified each model as non-susceptible (NS), moderately susceptible (MS), or highly susceptible (HS) to repolarization abnormalities (see Methods). Out of 568 models, 380 models (67%) were NS, 147 (26%) were MS and 41 (7%) were HS.

We investigated whether the susceptibility of models could be distinguished prior to current block by the control values of any of the seven biomarkers we used to calibrate the population. We found that the median control conditions biomarker values for all biomarkers except for V_m_ Time were significantly different (Mann-Whitney U-test, *p* < 0.001) between the sub-population of HS models and the rest of the population (Figure 5D). To determine whether control biomarker values were sufficient to predict which models were highly susceptible to repolarization abnormalities, we constructed a logistic regression model using the biomarkers of the population as predictor variables, to estimate the probability of each parameter set belonging to either the HS or non-HS groups. Over 1,000 iterations of 10-fold cross validation, the logistic regression model only achieved a 61% mean rate of correctly predicting HS models to be in the HS category. This low level of sensitivity suggests that the biomarkers used to calibrate the population are insufficient to predict model susceptibility prior to current block. This is likely due to the high level of overlap in biomarker distributions between the HS and the two other model classifications.

We then tested whether any of the nine conductances that were varied to create the population of models were significantly different between any of the three categories. Median G_Na_, G_CaL_, and G_NaK_ values were significantly different between all three categories of model (*p* < 0.05/27, Figure 5E). G_Na_ and G_CaL_ were larger in the higher susceptibility (MS and HS) categories, while G_NaK_ was smaller. G_NCX_ was also significantly different between the NS and HS categories, but not between the MS category and either of the other categories. Additionally, the range of G_NCX_ for all three categories spanned the full sampled range of the conductance, unlike G_Na_, G_CaL_, and G_NaK_ (Figure 5E). Therefore, we focused on G_Na_, G_CaL_, and G_NaK_ as potential contributors to repolarization abnormality susceptibility.

**Figure 6:**
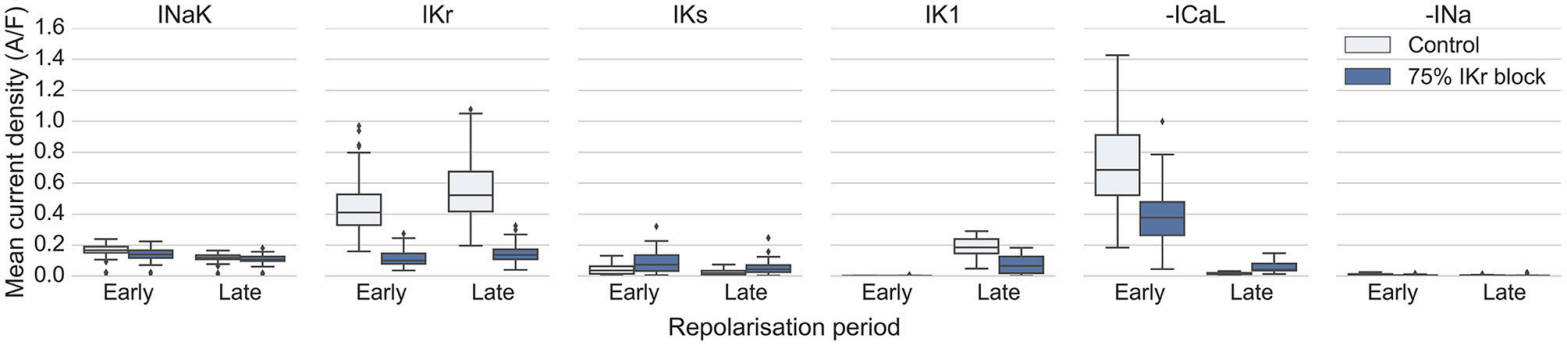
**Magnitude of I_**Na**_, I_**CaL**_, I_**NaK**_, I_**Kr**_, I_**Ks**_, and I_**K1**_ during repolarization for HS models in control conditions and under 75% I_**Kr**_ block**. Each current was averaged from the peak of the upstroke to APD40 (early) and from APD_40_ to APD_90_ (late), for each model.

Using the models in the HS category, we investigated the relative importance of I_Na_, I_CaL_, and I_NaK_, and repolarizing currents I_Kr_, I_Ks_ and I_K1_, during early and late periods of repolarization (we define early repolarization as from the AP peak to 40% repolarization, and late repolarization as from 40 to 90% repolarization). Figure 6 displays the average magnitudes of those six currents during each period of repolarization for the 41 HS models, under control conditions and 75% I_Kr_ block (data for NS and MS models are shown in Figure S4). Under control conditions I_Kr_ was the largest repolarizing current (mean current density from V_m_ Peak to APD_90_: 0.49 ± 0.21 A/F). Unexpectedly, I_NaK_ was the second largest repolarizing current overall (0.15 ± 0.05 A/F), substantially larger than I_Ks_ (0.04 ± 0.03 A/F), although I_K1_ was larger than I_NaK_ during late repolarization only (I_K1_: 0.18 ± 0.07 A/F, I_NaK_: 0.12 ± 0.04 A/F). During 75% I_Kr_ block, I_NaK_ became the largest repolarizing current (I_NaK_: 0.11 ± 0.04 A/F, I_Kr_ under 75% block: 0.03 ± 0.02 A/F). I_CaL_ was a major contributor of inward current during early repolarization under both control conditions and I_Kr_ block. During late repolarization the magnitude of I_CaL_ under 75% I_Kr_ block was increased by over 200% compared to control conditions (control: −0.05 ± 0.03 A/F, I_Kr_ block: −0.16 ± 0.01 A/F), which supports the importance of I_CaL_ reactivation in the generation of repolarization abnormalities. I_Na_ magnitude was not elevated under I_Kr_ block compared to control conditions (control: −0.007 ± 0.005 A/F, I_Kr_ block: −0.003 ± 0.004 A/F), suggesting it does not contribute to repolarization abnormality generation directly. However, we found that models with high G_Na_ correlated with models with low G_NaK_ (Figure 3C). Therefore, high G_Na_ may be compensatory for the increased intracellular Na^+^ in models with low G_NaK_ (mean intracellular Na^+^ was 16.4 ± 6.4 mM in HS models, compared with 8.9 ± 3.9 mM in the rest of the population), and the resulting decrease in inward Na^+^ driving force.

Overall, we identified low G_NaK_ and high G_CaL_ as key factors that increased susceptibility to repolarization abnormalities and distinguished highly susceptible models from the rest of the population, and found that I_NaK_ makes a key contribution to repolarization current, particularly under I_Kr_ block.

### Low G_NaK_ decreases repolarization reserve as well as increasing intracellular Na^+^

**Figure 7:**
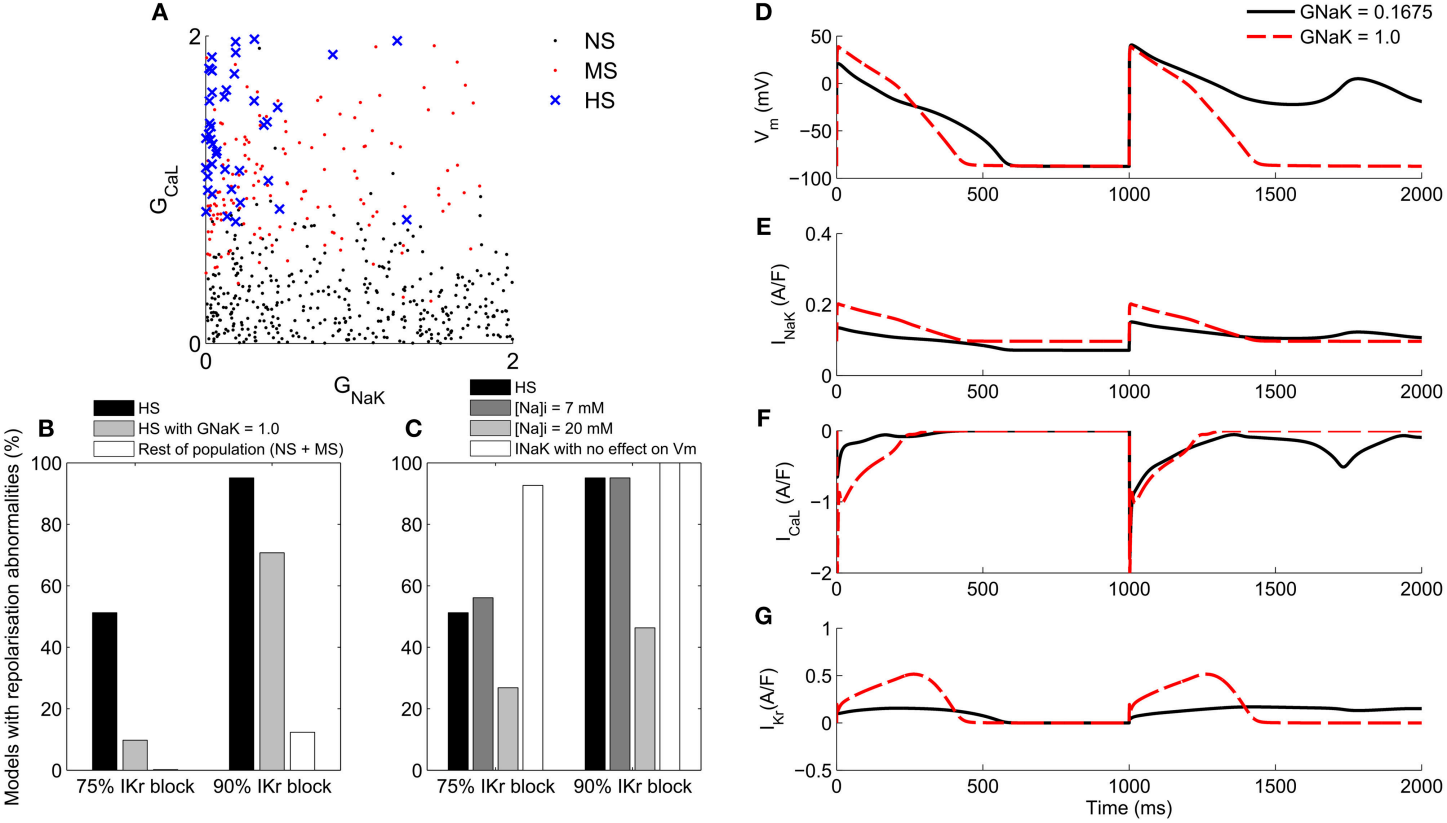
**Mechanisms of repolarization abnormality formation**. **(A)**: G_CaL_ and G_NaK_ values of models in the population classified by susceptibility to repolarization abnormalities. Black: NS. Red: MS. Blue crosses: HS. **(B)** Incidence of repolarization abnormalities in the HS sub-population (*n* = 41) at 75 and 90% I_Kr_ block compared to the same sub-population with G_NaK_ = 1.0, and to the rest of the population of models. **(C)** Incidence of repolarization abnormalities in the HS sub-population under different conditions. Left to right: baseline I_Kr_ block; with intracellular Na^+^ fixed to 7 mM; with intracellular Na^+^ fixed to 20 mM and intracellular K^+^ fixed to 145 mM; and with the electrogenic effect of I_NaK_ on V_m_ removed. **(D–G)** Voltage and selected ionic current traces showing abnormality occurrence in a representative model with original value of G_NaK_ scaling factor = 0.1675 (solid black) and with G_NaK_ increased to a scaling factor of 1.0 (red dashes).

A large majority (38/41) of the HS models in the population had G_NaK_ values of less than 0.5 times its value in the baseline ORd model, and values of G_CaL_ greater than 0.78 times the baseline value (Figure 7A). Repolarization abnormality occurrence across the HS models under 75 and 90% I_Kr_ block was 51 and 95% of models respectively (Figure 7B). After increasing G_NaK_ to the ORd model's baseline value (i.e., a scaling factor of 1.0) in each HS model, repolarization abnormality occurrence fell to 10 and 71% of models respectively. The reversion of an example HS model to a normal AP phenotype following increase of G_NaK_ in this way is shown in Figures 7D–G.

We hypothesized that the importance of G_NaK_ in determining susceptibility to repolarization abnormalities could be due to I_NaK_'s role in maintaining intracellular Na^+^, the significant contribution of I_NaK_ to repolarization reserve during I_Kr_ block (Figure 6), or a combination of both effects. We therefore investigated the effects of intracellular Na^+^ concentration and the electrogenic action of I_NaK_ individually, to evaluate their contributions to the development of repolarization abnormalities. To investigate how intracellular Na^+^ affected repolarization abnormality occurrence, we clamped its concentration to a physiologically normal value of 7 mM (the baseline concentration in the ORd model), and to an overloaded value of 20 mM, which is within the range reported in heart failure (Pieske et al., 2002) and for acute ischemia. For the 20 mM condition, we also clamped intracellular K^+^ to 145 mM to prevent rundown.

For intracellular Na^+^ clamped at 7 mM, repolarization abnormalities occurred in 56 and 95% of models at 75 and 90% I_Kr_ block respectively, close to the values for unclamped Na^+^. However, clamping intracellular Na^+^ at 20 mM and K^+^ at 145 mM caused a reduction in repolarization abnormalities to 27 and 46% of models at 75 and 90% I_Kr_ block respectively (Figure 7C). These results suggested that normal Na^+^ concentration alone was not sufficient to prevent repolarization abnormalities, and that elevated intracellular Na^+^ may make an important contribution to repolarization reserve and decrease occurrence of repolarization abnormalities by increasing the outward current through I_NaK_.

To test whether the outward current provided by I_NaK_ itself was an important contributor to repolarization reserve during I_Kr_ block, we removed the electrogenic effects of I_NaK_ on membrane potential, while still allowing ion transport to occur normally. Without the electrogenic contribution of I_NaK_, repolarization abnormalities occurred in 93% of low G_NaK_ HS models following 75% I_Kr_ block, and in 100% of models following 90% I_Kr_ block (Figure 7C). This large increase in abnormalities when only the electrogenic component of I_NaK_ was removed, even in models with a low baseline G_NaK_, combined with the large relative magnitude of I_NaK_ during repolarization shown in Figure 6, provides evidence supporting an important role for I_NaK_ as a contributor to repolarization reserve in humans during I_Kr_ block, over a wide range of different conductance profiles.

### Effects of hypokalemia and rapid pacing on repolarization

**Figure 8:**
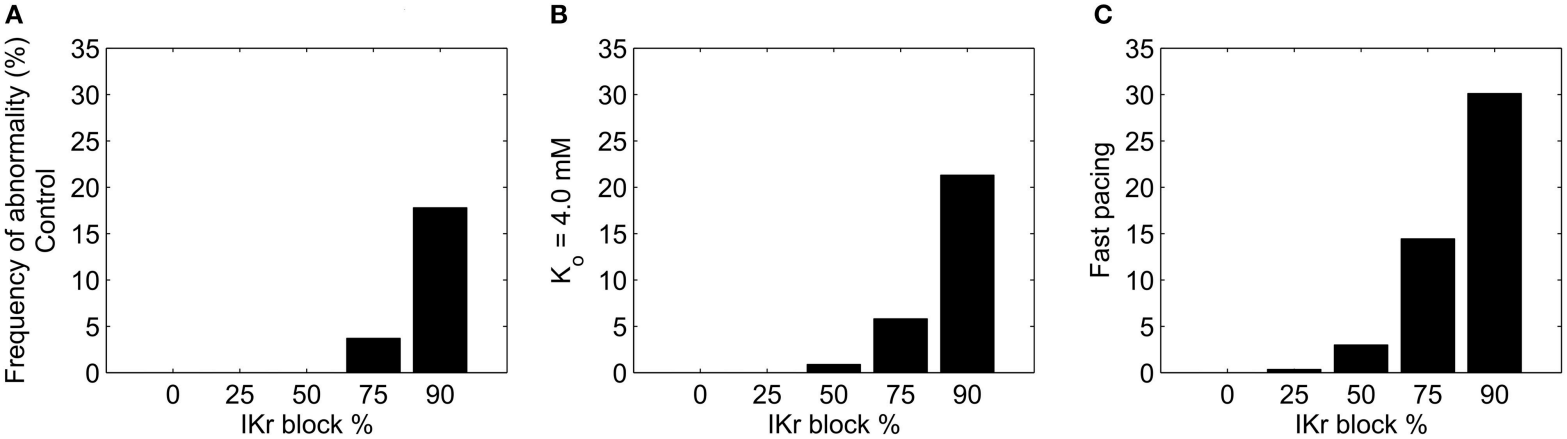
**Incidence of repolarization abnormalities in the population of models under different levels of I_**Kr**_ block, following changes to extracellular potassium and intracellular calcium concentrations. (A)** Control conditions ([K^+^]_o_ = 5.4 mM, 1 Hz pacing). **(B)** Reduced extracellular potassium concentration ([K^+^]_o_ = 4.0 mM). **(C)** Rapid pacing (2.5 Hz) to increase Ca^2+^ loading, followed by sudden decrease to 1 Hz.

Hypokalemia and Ca^2+^ loading are both known to increase the likelihood of afterdepolarization generation (Sato et al., 2010; Weiss et al., 2010; Despa and Bers, 2013). We investigated how much each phenomenon altered the incidence of repolarization abnormalities in the population of models under I_Kr_ block (Figure 8). Mild hypokalemia was simulated by reducing extracellular potassium from 5.4 to 4.0 mM in each model. Each model in the population was simulated under these conditions at five levels of I_Kr_ block: 0/25/50/75/90%.

Reducing extracellular K^+^ alone was not sufficient to generate repolarization abnormalities, but, combined with I_Kr_ block it resulted in a small increase in the number of models that displayed abnormalities relative to control K^+^ levels (Figure 8B).

To investigate whether susceptibility was increased by Ca^2+^ loading, we used rapid pacing at 2.5 Hz, which is known to cause Ca^2+^ loading (Maruyama et al., 2010), followed by a reduction in pacing rate to 1 Hz for the final two beats of each simulation, to match the pacing rate to the other simulations in this study. This protocol resulted in 52% of models displaying an average increase in intracellular Ca^2+^ of 50% or more compared to pacing at 1 Hz. The final two beats of each simulation showed no development of afterdepolarizations across the population under control conditions. However, pacing at 2.5 Hz lead to increased repolarization abnormality occurrence following I_Kr_ block, as shown in Figure 8C. Afterdepolarization occurrence across the population increased from 4 to 14% of models at 75% I_Kr_ block, and from 18 to 30% at 90% I_Kr_ block.

## Discussion

### Main findings

In this study, a population of human ventricular cardiomyocytes, calibrated using electrophysiological recordings to yield normal APs under control conditions, provides a quantitative understanding of the relative importance of ionic currents in the occurrence of repolarization abnormalities under ionic drug block. Our main findings include:

1. I_Kr_ block was necessary for repolarization abnormalities to occur. No model in the population displayed repolarization abnormalities in any of the simulations in this study where I_Kr_ was not blocked. This result is supported by the inclusion of simulations with simultaneous 90% block of the repolarizing K^+^ currents I_Ks_ and I_K1_, and the presence of models in our population with very low values of G_Kr_ (Figure 3C). This finding highlights the particular importance of I_Kr_ in human ventricular cardiomyocytes, and the unique role of I_Kr_ block in destabilizing repolarization.
2. Under I_Kr_ block, I_NaK_ and also I_CaL_ are the most important determinants of repolarization abnormality susceptibility. The importance of G_NaK_ is a surprising finding, as the focus of investigation into the mechanisms of ventricular repolarization abnormalities has been on the roles of K^+^ and Ca^2+^ currents such as I_Kr_, I_Ks_, and I_CaL_ (Roden and Abraham, 2011), and not on I_NaK_. Reduced I_NaK_ is common in disease such as ischaemia and heart failure (Schwinger et al., 1999; Fuller et al., 2003), is known to contribute to delayed afterdepolarization (DAD) formation (Rosen et al., 1973; Ferrier, 1976; Rosen, 1985), and can increase intracellular Ca^2+^ through Na^+^ overload (Despa and Bers, 2013). However, I_NaK_ was not considered in previous studies to have a major role in generating repolarization abnormalities during the AP (e.g., EADs). Identification of high G_*CaL*_ was expected, based on previous experimental and computational studies highlighting the importance of I_CaL_ for EAD generation (January and Riddle, 1989; Sims et al., 2008; Vandersickel et al., 2014).
3. Our quantitative analysis shows that I_NaK_ is the main contributor to human repolarization current following 75% I_Kr_ block, and the second largest contributor under control conditions, after I_Kr_. Its contribution to repolarization reserve was larger than that of I_Ks_ (Figure 6).
4. I_NaK_'s low electrogenic contribution to repolarization reserve was a necessary component of the mechanism that caused repolarization abnormalities. Na^+^ overload alone was not sufficient to explain how low G_NaK_ caused repolarization abnormalities. Clamping Na^+^ to a physiological level (7 mM) had little effect on abnormality occurrence, while clamping Na^+^ at an overloaded level (20 mM) decreased abnormality occurrence. Removing the electrogenic effect of I_NaK_ while retaining its ability to transport ions caused a large increase in repolarization abnormalities. Thus, repolarization abnormality occurrence was higher in low G_NaK_ models because of changes in repolarizing current and ionic concentrations, and not Na^+^ overload alone.

### Important role of I_NaK_ in human repolarization reserve and drug-induced repolarization abnormalities

Unexpectedly, we found that I_NaK_ was an important component of repolarization reserve, specifically, that it made the second largest overall contribution to repolarizing current after I_Kr_. While I_Kr_ inhibition and I_CaL_ availability were sufficient for repolarization abnormalities to occur in the strongest drug block scenarios (i.e., 90% I_Kr_ block combined with block of either I_Ks_ or I_K1_), when we analyzed the 41 models in the HS sub-population, we found that G_NaK_ was less than 50% of its baseline value in 38/41 of these models (Figure 7A). Even in these models, I_NaK_ was the largest contributor to repolarization current after I_Kr_, and of greater magnitude than I_Ks_ following 75% I_Kr_ block. A number of disease conditions are associated with reduced expression of the Na^+^/K^+^ pump (Bueno-Orovio et al., 2014), including ischemia (Fuller et al., 2003), heart failure (Xu et al., 1996; Schwinger et al., 1999), and additional comorbidities such as diabetes (Bossuyt et al., 2005). The pump is also selectively blocked by cardiac glycosides, such as digoxin, digitoxin and digitalis (Suhail, 2010). Reports of the effects of reduced G_NaK_ have focused on the role of the pump in maintaining Na^+^ homeostasis, with elevation of intracellular Na^+^ concentration linked to compromised I_NaK_ (Wasserstrom and Aistrup, 2005). Our results identified G_NaK_ as an important determinant of repolarization reserve across a wide range of conductance profiles, and suggest that reduction of repolarization reserve could be a further pro-arrhythmic effect of reduced I_NaK_.

As well as being reduced in disease conditions, I_NaK_ is inhibited by a range of drugs, including multichannel blockers and cardiac glycosides. For example, amiodarone, a commonly used anti-arrhythmic compound that affects multiple channels, also inhibits I_NaK_ (Gray et al., 1998). Furthermore, there is evidence that cardiac glycosides can reduce trafficking of the hERG channel, which conducts I_Kr_ in humans, and in particular, that digitoxin could cause a significant reduction in hERG trafficking at therapeutic concentrations (Wang et al., 2007). Another cardiac glycoside, digoxin, has been associated with increased risk of sudden cardiac death (Niemeijer et al., 2015). Therefore, there is a need for further experimental investigation to clarify the role of I_NaK_ in ventricular repolarization, particularly following I_Kr_ block. In addition, as the ORd model currently represents our most comprehensive quantitative model of human ventricular cardiomyocyte repolarization, experimental testing of this study's predictions will be important for determining if the ORd model accurately represents the role of I_NaK_ in repolarization, or if the current model needs to be updated.

### AP biomarkers could not distinguish normal and abnormal models

Within the population of models a wide variety of conductance profiles produced viable APs (Figure 3A), far from the original conductances of the baseline ORd model. This is consistent with results from previous studies showing the robustness of APs to changes in ionic conductances (Marder and Taylor, 2011; Sarkar and Sobie, 2011; Sánchez et al., 2014; Passini et al., 2016; Zhou et al., 2016). Therefore, all models in the population displayed normal APs with important AP biomarkers within the experimental physiological range. However, across the population there were large differences in models' responses to current block. Even under high levels of I_Kr_ block, most models did not develop repolarization abnormalities (Figures 5A–C), but almost all models did exhibit substantial APD prolongation (Figure S3B). Therefore, while strong I_Kr_ block alone is sufficient to cause AP prolongation, it is not sufficient to cause repolarization abnormalities, and also requires an additional mechanism, such as the combination of high I_CaL_ and low I_NaK_. Further, through a logistic regression model, we found that different levels of susceptibility to drug-induced repolarization abnormalities could not be identified prior to current block using standard AP biomarkers and control conditions simulations alone. The logistic regression model could, on average, only correctly assign 61% of the HS models to the HS category. However, combining classification techniques from machine learning and recordings from additional experimental conditions could potentially discover combinations of key features that are more predictive of response to drug block.

### Future directions

In this study, we focused on variability of channel densities, and therefore sarcolemmal conductances, as an important source of variability because of the wide array of biological phenomena known to alter ion channel expression and trafficking, and because conductance alteration through block is the primary mechanism through which many pharmaceutical compounds act on the heart. We have shown that variation in sarcolemmal conductances alone is sufficient to produce drug-induced repolarization abnormalities in models with normal control APs and APDs. However, as ion channel kinetics can vary significantly between individuals, e.g., through genetic mutations (Marban, 2002), future studies could investigate how variation in channel kinetics interacts with conductance variability, e.g., for inherited channelopathies associated with long QT syndrome. For studies investigating phenomena that are known to increase the current through particular channels (e.g., beta-adrenergic stimulation), increasing the range of sampled conductance values for those particular currents could also be useful.

The experimental recordings used in this study, and therefore the sources of AP biomarker variability used to calibrate the population of models, were recorded from endocardial tissue in the right ventricle. Electrophysiological properties vary intramurally and between left and right ventricles (Bueno-Orovio et al., 2012), and these additional sources of electrophysiological variability were not considered in this study. However, we believe the wide ranges of AP biomarker values and conductance profiles considered here mitigate this limitation, particularly relative to using a single model of ventricular electrophysiology.

Although this was not the focus of our investigations, reduced I_NaK_ has been linked to DAD occurrence (Rosen et al., 1973; Rosen, 1985; Despa and Bers, 2013). DADs were not observed in any model in the population using the stimulation protocols in our simulations, although we did not specifically design simulation protocols to generate them. Therefore, specific simulation protocols and possibly alterations in model structure, particularly in the calcium sub-system, may be required for these investigations.

Even under heavy I_Kr_ block, only a minority of models in the population displayed abnormalities, and these models occupied a limited parameter regime. It is therefore likely that EAD formation *in vivo* requires the combined action of multiple destabilizing factors, as in healthy tissue electrotonic coupling suppresses repolarization abnormalities unless they occur simultaneously in a large number of coupled cells (Xie et al., 2010). Electrotonic coupling would also likely prevent the complete repolarization failure that occurred in some models under high I_Kr_ block from occurring *in vivo*. However, in diseased or damaged tissue, reduced cellular coupling, combined with increased structural heterogeneity could potentially overcome electrotonic coupling (Shaw and Rudy, 1997; Xie et al., 2010), or even cause electrotonic current to become a generative mechanism for EAD development (Dutta et al., 2016). This tissue-level destabilization could increase the range of ionic profiles that are vulnerable to developing repolarization abnormalities and reduce the level of drug block required to initiate them. Therefore, understanding how tissue heterogeneity interacts with variability in conductances and drug block to influence repolarization abnormality formation is an important topic for future investigations.

## Author contributions

Conceived and designed study: OB, AB, and BR. Acquired experimental recordings: LV and AV. Performed simulations and analyzed data: OB. Drafted manuscript: OB, AB, and BR. All authors contributed to critical revision of the manuscript and approved the final version to be published.

## Funding

This work was supported by an Engineering and Physical Sciences Research Council-funded Systems Biology Doctoral Training Centre studentship and Doctoral Prize (OB), the 2014 National Centre for the 3Rs Prize (OB) and a Welcome Trust Senior Research Fellowship in Basic Biomedical Science to BR (100246/Z/12/Z) (AB, LV, AV, and BR).

### Conflict of interest statement

The authors declare that the research was conducted in the absence of any commercial or financial relationships that could be construed as a potential conflict of interest.
