# Corrigendum: The Electrogenic Na^+^/K^+^ Pump Is a Key Determinant of Repolarization Abnormality Susceptibility in Human Ventricular Cardiomyocytes: A Population-Based Simulation Study

**DOI:** 10.3389/fphys.2017.00954

**Published:** 2017-11-17

**Authors:** Oliver J. Britton, Alfonso Bueno-Orovio, László Virág, András Varró, Blanca Rodriguez

**Affiliations:** ^1^Department of Computer Science, University of Oxford, Oxford, United Kingdom; ^2^Department of Pharmacology and Pharmacotherapy, Faculty of Medicine, University of Szeged, Szeged, Hungary

**Keywords:** human, repolarization, cardiac electrophysiology modeling, variability, sodium-potassium pump, Na^+^/K^+^ pump

In our original article, the list of funders was not complete. Therefore, the funding statement:

“This work was supported by an Engineering and Physical Sciences Research Council-funded Systems Biology Doctoral Training Centre studentship and Doctoral Prize (OB), the 2014 National Centre for the 3Rs Prize (OB) and a Welcome Trust Senior Research Fellowship in Basic Biomedical Science to BR (100246/Z/12/Z) (AB, LV, AV, and BR).”

should instead read:

“This work was supported by an Engineering and Physical Sciences Research Council-funded Systems Biology Doctoral Training Centre studentship and Doctoral Prize (OB), the 2014 National Centre for the 3Rs Prize (OB), a Welcome Trust Senior Research Fellowship in Basic Biomedical Science to BR (100246/Z/12/Z) (AB, LV, AV, and BR) and by the National Research, Development and Innovation Office (NKFI/OTKA NN-109904(LV and AV).”

The authors apologize for this error and state that this does not change the scientific conclusions of the article in any way.

## Conflict of interest statement

The authors declare that the research was conducted in the absence of any commercial or financial relationships that could be construed as a potential conflict of interest.

