# Supplementary material for: The Electrogenic Na+/K+ Pump Is a Key Determinant of Repolarization Abnormality Susceptibility in Human Ventricular Cardiomyocytes: A Population-Based Simulation Study
Source: Front Physiol. 2017 May 5;8:278. doi: 10.3389/fphys.2017.00278 (PMC5418229; doi:10.3389/fphys.2017.00278)
Supplement: Supplementary file 1 [file DataSheet1.DOCX]

Supplementary Material

**The Electrogenic Na^+^/K^+^ pump is a Key Determinant of Repolarization Abnormality Susceptibility in Human Ventricular Cardiomyocytes: A Population-Based Simulation Study**

**Oliver J. Britton*, Alfonso Bueno-Orovio , László Virág , András Varró , Blanca Rodriguez**

*** Correspondence:** Oliver Britton: oliver.britton@cs.ox.ac.uk

**1. Supplementary Methods**

**S1. Choice of the O’Hara-Rudy model**

The O'Hara-Virag-Varro-Rudy (ORd) model (O’Hara et al., 2011) of the human ventricular cardiomyocyte was used as the baseline model for our investigations, as it is one of the most recent, widely used and extensively tested models of the human ventricular cardiomyocyte, and is particularly well-suited for studying human ventricular repolarization, as key currents involved in repolarization and EAD formation (including I_Kr_, I_Ks_, I_K1_ and I_CaL_) are parameterized using data exclusively from undiseased human ventricular cardiomyocytes.

The ORd model has been used in many recent investigations into cardiac arrhythmia mechanisms. It was developed primarily from a large dataset of human-specific voltage clamp and microelectrode recordings from >140 hearts, performed by the same laboratory that recorded the data used in this study. This direct link to human cardiac electrophysiology has led to the model being used in over 30 recent investigations into cardiac arrhythmia mechanisms (Table S1). It has been used to investigate a diverse range of phenomena in human cardiac cellular electrophysiology including: the effects of potassium channel mutations on repolarization; prediction of channel-blocking drug effects on the AP and calcium transient; cellular mechanisms of alternans and EADs; and the consequences of ion channel remodelling and changes to ionic homeostasis due to heart failure, Brugada syndrome, and other cardiac diseases. It has also been used as the underlying model of cellular electrophysiology in multiscale 1D and 3D ventricular models to investigate tissue-level effects such as electromechanical coupling and transmural heterogeneity.

We modified the fast Na^+^ current's inactivation gate as in Passini et al. (2016) to increase robustness of AP upstroke generation at hyperpolarised resting potentials. Thus, the formulation for the inactivation (h) gate that we use in this study is:

$h_{ss}=\frac{1}{1+e^{{(V}_{m}+78.4893)/6.2248}}$,

$h_{ssp}=\frac{1}{1+e^{(V_{m}+84.689)/6.2248}}$,

where V_m_ is the membrane potential, h_ss_ is the steady state value of the h gate, and h_ssp_ is the steady state value of the phosphorylated h gate.

**S2. AP biomarkers for calibration of populations of human ventricular cardiomyocyte models**

The following AP biomarkers (Figure 1) were used in this study:

Peak membrane potential (V_m_ Peak) - the peak value of the membrane potential following stimulus.

Peak membrane time (V_m_ Time) - the time, measured from the beginning of the pacing cycle, at which the peak membrane potential occurs.

Resting membrane potential (RMP): The mean membrane potential during the final 50 ms of the pacing cycle.

AP duration at 40/50/90% repolarisation (APD_40/50/90_) - The time from the beginning of the stimulus to the first point where the membrane potential reaches a given percentage of full repolarisation, defined by the difference between peak and resting membrane potential.

Triangulation_90-40_ - the difference between APD_90_ and APD_40_.

Additionally, the final two simulated beats of each model, paced at 1 Hz, were checked for alternans. Alternans were classed as a difference in APD_90_ between the final two beats of a simulation of at least 5 ms. If alternans were detected, the model was discarded. This criterion was chosen to ensure no models displayed abnormalities under control conditions at a moderate pacing rate, prior to block of any currents.

**S3. Stimulation protocol**

Unless specified otherwise, in all simulations in this study models were paced at 1 Hz, for 1000 s to allow each model to equilibrate. As the experimental data used in this study was recorded from small tissue samples (Jost et al., 2005), rather than isolated cells, we adopted the biphasic stimulus protocol described by Livshitz et al. (2009), in order to simulate the electrotonic interactions between coupled myocytes. In the biphasic protocol, the standard inward square wave of current injection is followed by an outward square wave with duration equal to the period between the end of the inward stimulus and the beginning of the next pacing cycle. The amplitude of this outward component is set so that the net injection of stimulus current into the cell, per stimulus, is 0. This reflects a key electrophysiological difference between isolated cardiomyocytes and small tissue preparations - in the latter the charge injected by the stimulus current can flow into the surrounding tissue. By using a biphasic stimulus we aimed to incorporate the current sink property of cardiac tissue into our cellular models.

The final two pacing cycles of each simulation on each model were saved and used to determine biomarker values for that simulation and model, and for detection of repolarization abnormalities.

**S4. Simulations**

Simulations were carried out on a desktop computer running a Linux operating system. The CVODE adaptive time step ODE solver library was used to solve the model equations using software described in Pitt-Francis et al. (2009), and data analysis was carried out in MATLAB (MathsWorks Inc., Natick, MA).

**S5. Conductances values in the calibrated population of models.**

Table S3 summarizes the conductances values if all human ventricular models included in the population calibrated with experimental data. Certain pairs of conductances display moderate to weak correlations with one another, which also constrains the observed parameter distribution of the population. Pairs of ionic conductances with significant correlations are shown with their correlation coefficients in Figure 3C-J. We used partial correlation coefficients to give a measure of correlation between parameters after accounting for the effects of the other varied parameters. We found moderate negative correlation between G_Na_ and G_NaK_, and between G_Kr_ and G_K1_. There were weak positive correlations between G_Na_ and G_CaL_, G_Na_ and G_NCX_, G_Kr_ and G_NaL_, and G_Kr_ and G_NCX_. However, most conductances are not correlated with one another and a wide range of conductance profiles give behaviour consistent with experiment.

**S6. Comparison of the uses of populations of human cardiomyocyte models in studies of arrhythmia mechanisms.**

Two other recent studies have used populations of models with the ORd model as a baseline to investigate different areas of cardiac electrophysiology (Zhou et al., 2016; Passini et al., 2016). We describe them briefly here to illustrate the differences and similarities between different studies that address different research questions using populations of models with the ORd model as a baseline.

Zhou et al. investigated how variability in ionic conductances influenced the occurrence of alternans and differentiated between different alternans types. A population-based approach was used to investigate how different types of alternans formed, based on the underlying ionic substrate, using action recovery interval data from 41 human ventricles to calibrate the population. This study used the ORd model due to its detailed calcium sub-system, which was important for studying the differences between voltage-driven and calcium transient-driven alternans, and its human-specificity, to allow consistency with the action recovery interval recordings. Unlike the present study, calcium sub-system parameters were varied along with sarcolemmal channel densities, due to the importance of calcium dynamics for the development of alternans.

Passini et al. studied how variability in ionic currents interacted with the electrophysiological remodelling induced by Hypertrophic Cardiomyopathy (HCM), a genetic disorder that causes remodelling in both myocardial structure and electrophysiology. This study used the ORd model to develop both undiseased and HCM-specific populations of models, based on data characterising the ionic remodelling of currents after HCM and both AP and calcium transient recordings. The HCM population was used to investigate how remodelling by HCM could lead to increased occurrence of repolarisation abnormalities, and to identify combinations of ion channel block that were more effective at reversing abnormality occurrence than block of any single channel alone. The initial control population was developed with variation of +/- 50% variation in conductances from the baseline ORd values, less than the ranges used in this study and the study by Zhou et al. which used +/- 100% variation from baseline values to construct their populations The populations constructed in Zhou et al. and the present study. This was because Passini et al.’s population was designed to represent substantial but not pathological levels of variability that could then be modified to represent myocytes from hearts with HCM. In contrast, Zhou et al. and the present study aimed to investigate a wide range of plausible ionic conductance profiles, including those that might only be found in diseased hearts or under sustained drug block.

**2. Supplementary Tables**

**Table S1:** Studies that reported using the ORd model for investigations of human ventricular electrophysiology.

| **Research area** | **Study references** |
| --- | --- |
| Potassium channel mutations (IKr/IKs/IK1) | **[7-16]** |
| Mechanisms and prediction of channel blocking drug effects | **[17-25]** |
| Contractility | **[26, 27]** |
| Disease-induced ionic remodelling | **[2, 27-31]** |
| Electrophysiological gender differences | **[32, 33]** |
| Mechanisms of EADs | **[2, 34]** |
| Mechanisms of alternans | **[6]** |
| Effects of beta-adrenergic stimulation | **[1, 15, 35]** |

**Table S2**: Calibration ranges of AP biomarkers from human ventricular AP recordings (n=62).

| Biomarker | Minimum | Maximum |
| --- | --- | --- |
| V_m_ Peak (mV) | 7.3 | 39.6 |
| V_m_ Time (ms) | 3.1 | 14.0 |
| APD_40_ (ms) | 84.9 | 323.6 |
| APD_50_ (ms) | 106.6 | 349.4 |
| APD_90_ (ms) | 178.1 | 442.7 |
| Triangulation_90-40_ (ms) | 61.2 | 153.0 |
| RMP (mV) | -94.4 | -78.5 |

**Table S3**: Parameter values for the 568 models in the accepted population of models. Rows correspond to models and columns to conductances in the following order: G_Na_, G_NaL_, Gto, G_Kr_, G_Ks_, G_K1_, G_NCX_, G_NaK_, G_CaL_. Each conductance is given as a scaling factor of the value of that conductance in the ORd model in the default endocardial configuration.

| Index | GNa | GNaL | GTo | GKr | GKs | GK1 | GNCX | GNaK | GCaL |
| --- | --- | --- | --- | --- | --- | --- | --- | --- | --- |
| 1 | 0.59938 | 0.14012 | 0.55085 | 1.0684 | 0.24308 | 0.78398 | 1.6252 | 0.061444 | 0.49282 |
| 2 | 1.5981 | 1.8031 | 1.3544 | 1.1785 | 0.047627 | 0.41862 | 0.53144 | 0.030776 | 0.353 |
| 3 | 0.68212 | 0.84429 | 1.9738 | 1.1782 | 1.7356 | 0.38101 | 1.0179 | 1.1174 | 0.33193 |
| 4 | 0.49983 | 1.9395 | 1.8217 | 0.76775 | 0.67166 | 1.1916 | 0.60196 | 1.188 | 0.17039 |
| 5 | 0.6746 | 1.2422 | 1.8771 | 0.81135 | 1.385 | 0.64776 | 0.7505 | 0.79975 | 0.36721 |
| 6 | 0.72306 | 0.78868 | 1.3576 | 0.38752 | 0.65134 | 1.2742 | 1.3652 | 0.37881 | 1.4206 |
| 7 | 1.0435 | 1.4926 | 1.8444 | 1.5049 | 1.9382 | 0.62953 | 1.1741 | 0.11202 | 1.1621 |
| 8 | 0.64816 | 1.4088 | 1.8068 | 1.5437 | 1.7321 | 0.39858 | 1.299 | 0.87489 | 1.4925 |
| 9 | 0.68833 | 0.87159 | 1.9078 | 1.9323 | 0.41984 | 0.19581 | 1.6526 | 0.1828 | 0.12713 |
| 10 | 0.31766 | 1.6058 | 1.712 | 0.59837 | 1.4169 | 0.98881 | 1.1601 | 0.7748 | 0.002378 |
| 11 | 0.41687 | 0.53015 | 1.8961 | 1.1721 | 0.053041 | 1.0656 | 0.01384 | 0.22144 | 0.29169 |
| 12 | 0.63105 | 0.18072 | 0.065883 | 0.73737 | 1.298 | 1.0767 | 0.88355 | 1.7189 | 0.25298 |
| 13 | 0.67572 | 1.7581 | 0.93184 | 1.3312 | 1.1114 | 0.87487 | 1.31 | 1.2101 | 0.59995 |
| 14 | 0.58545 | 1.9933 | 0.72039 | 1.344 | 1.5734 | 0.40891 | 1.5491 | 1.9317 | 0.20689 |
| 15 | 0.81433 | 0.93728 | 0.70161 | 1.0033 | 1.5416 | 1.8029 | 1.4552 | 0.35229 | 0.74927 |
| 16 | 0.50444 | 1.4359 | 1.2924 | 0.37958 | 1.8108 | 1.2659 | 0.82951 | 1.8418 | 0.12024 |
| 17 | 0.51418 | 1.0375 | 1.8881 | 1.1253 | 0.10398 | 0.075529 | 0.098881 | 1.6399 | 1.1601 |
| 18 | 0.36696 | 0.17001 | 0.028252 | 0.89782 | 1.7306 | 1.6704 | 0.21275 | 1.8651 | 0.26675 |
| 19 | 0.57179 | 0.081433 | 0.88767 | 0.44436 | 1.0454 | 1.447 | 0.77088 | 1.7139 | 0.15843 |
| 20 | 0.58319 | 0.71726 | 0.89584 | 1.0205 | 1.2418 | 0.98635 | 0.14321 | 0.15245 | 0.59959 |
| 21 | 0.92643 | 1.6688 | 1.8552 | 1.4451 | 0.036584 | 0.80944 | 1.9318 | 0.42937 | 0.22274 |
| 22 | 0.4644 | 1.1885 | 1.9202 | 1.0206 | 0.17259 | 0.7213 | 0.091408 | 0.46563 | 0.26475 |
| 23 | 0.43144 | 1.698 | 0.46716 | 1.1642 | 1.4442 | 1.5495 | 0.21587 | 0.33771 | 0.23463 |
| 24 | 1.0633 | 1.5228 | 1.1959 | 0.74226 | 1.9769 | 1.0036 | 0.4952 | 0.085828 | 0.45307 |
| 25 | 0.681 | 1.8307 | 1.6915 | 1.2495 | 1.3567 | 0.3654 | 0.58514 | 1.7687 | 0.47386 |
| 26 | 0.66305 | 1.2721 | 0.97298 | 1.1293 | 1.9026 | 1.4084 | 1.2835 | 1.0123 | 0.7426 |
| 27 | 0.28247 | 0.10617 | 0.10108 | 0.48951 | 1.5969 | 1.8407 | 0.10204 | 0.3249 | 0.10611 |
| 28 | 0.64194 | 0.58944 | 1.2166 | 0.86778 | 1.2231 | 0.8921 | 0.49811 | 0.63574 | 0.21345 |
| 29 | 0.2896 | 1.1175 | 0.003248 | 0.26287 | 0.57779 | 1.7818 | 0.003981 | 0.53223 | 0.008594 |
| 30 | 0.7175 | 0.67096 | 1.6185 | 0.78299 | 0.17699 | 0.79426 | 1.11 | 0.53219 | 1.0384 |
| 31 | 1.6934 | 0.38081 | 0.37254 | 1.7399 | 1.0075 | 0.45563 | 1.3632 | 0.027864 | 1.4183 |
| 32 | 0.60524 | 1.4384 | 0.67694 | 1.01 | 0.8312 | 0.84377 | 0.96376 | 0.61052 | 1.3062 |
| 33 | 0.55935 | 0.044958 | 1.8269 | 0.32074 | 0.74302 | 1.0588 | 0.37949 | 1.9976 | 0.027326 |
| 34 | 0.37198 | 1.2785 | 1.1273 | 1.7439 | 1.4191 | 0.49047 | 1.9335 | 0.73294 | 0.33919 |
| 35 | 0.5621 | 1.0511 | 0.86377 | 0.85521 | 0.18132 | 1.8948 | 1.2543 | 0.65079 | 0.99142 |
| 36 | 0.56738 | 0.49406 | 0.34223 | 0.86216 | 1.4484 | 1.1048 | 1.5671 | 0.052313 | 0.013428 |
| 37 | 0.48329 | 1.5417 | 0.3616 | 0.61786 | 0.53697 | 0.34751 | 0.49145 | 0.65446 | 0.39712 |
| 38 | 0.72778 | 0.60186 | 0.66305 | 0.73982 | 1.3095 | 0.82919 | 1.0322 | 0.39166 | 0.10219 |
| 39 | 0.51508 | 1.7148 | 1.901 | 0.94861 | 0.25849 | 1.9547 | 0.067697 | 0.012832 | 0.58215 |
| 40 | 0.67459 | 1.8047 | 1.8279 | 1.2427 | 0.3377 | 0.6715 | 0.71468 | 0.079358 | 0.98236 |
| 41 | 0.89795 | 0.95733 | 1.509 | 1.0251 | 0.78562 | 0.67562 | 0.83346 | 0.16969 | 0.16462 |
| 42 | 0.61783 | 1.1781 | 1.9986 | 1.0695 | 1.781 | 0.29212 | 1.762 | 0.74956 | 1.6026 |
| 43 | 0.66414 | 0.87965 | 1.2822 | 1.0154 | 1.0431 | 1.116 | 1.1607 | 0.93436 | 0.34306 |
| 44 | 1.0555 | 1.3599 | 1.2086 | 0.84332 | 1.3852 | 0.31592 | 0.50727 | 0.065417 | 0.559 |
| 45 | 1.1874 | 0.88738 | 0.75527 | 0.70867 | 1.213 | 1.154 | 1.4836 | 0.013306 | 0.99693 |
| 46 | 0.63363 | 0.32634 | 0.98131 | 0.89984 | 1.3196 | 0.037067 | 0.35089 | 0.39299 | 0.62106 |
| 47 | 0.43518 | 1.9256 | 1.0995 | 0.37475 | 1.7426 | 0.97164 | 1.7524 | 1.3983 | 0.1546 |
| 48 | 1.7793 | 1.0825 | 0.86554 | 1.1276 | 1.1033 | 1.2168 | 1.307 | 0.002357 | 0.45826 |
| 49 | 0.55255 | 0.65223 | 0.35217 | 1.0413 | 1.4952 | 0.92277 | 1.4935 | 0.46084 | 0.67306 |
| 50 | 0.6703 | 1.5884 | 1.7205 | 0.60739 | 0.61461 | 0.62978 | 0.21239 | 0.39967 | 1.4413 |
| 51 | 0.77302 | 1.4729 | 0.62471 | 0.6711 | 0.79031 | 0.92514 | 0.73554 | 0.47658 | 0.006628 |
| 52 | 0.7318 | 1.8033 | 0.44471 | 1.5683 | 1.435 | 0.097485 | 1.3559 | 0.62187 | 0.47173 |
| 53 | 0.4259 | 0.35669 | 1.2022 | 0.53824 | 0.53648 | 1.9095 | 1.7216 | 0.82886 | 0.050253 |
| 54 | 0.64259 | 1.5573 | 1.4474 | 1.0547 | 0.59095 | 0.84947 | 0.3301 | 0.63945 | 0.75922 |
| 55 | 0.69975 | 0.10785 | 1.5084 | 1.0296 | 0.78309 | 0.68748 | 1.6797 | 1.8243 | 0.24308 |
| 56 | 0.49113 | 1.4168 | 0.078897 | 1.1532 | 0.12797 | 1.3452 | 0.094752 | 1.2978 | 0.89005 |
| 57 | 1.4901 | 1.9726 | 0.2154 | 1.2423 | 0.057702 | 0.62783 | 1.8551 | 0.040723 | 1.8629 |
| 58 | 1.2146 | 1.492 | 0.90471 | 0.76162 | 1.3743 | 0.51174 | 0.56042 | 0.026403 | 1.7858 |
| 59 | 0.70787 | 1.7601 | 0.07286 | 0.60011 | 0.19501 | 0.93225 | 1.441 | 0.19542 | 0.7924 |
| 60 | 0.69154 | 0.29564 | 1.0901 | 0.63122 | 0.79882 | 1.2868 | 1.0458 | 0.66081 | 0.81225 |
| 61 | 1.1051 | 0.26307 | 0.34695 | 1.6113 | 0.94241 | 0.58412 | 1.2959 | 0.027191 | 0.96314 |
| 62 | 0.42601 | 0.12115 | 0.23031 | 1.1357 | 1.7439 | 1.1415 | 1.712 | 0.16723 | 0.19534 |
| 63 | 0.5148 | 0.56529 | 0.43519 | 1.3959 | 0.29089 | 0.39627 | 1.51 | 0.28144 | 1.3087 |
| 64 | 0.50769 | 1.0421 | 0.27081 | 1.2902 | 1.0629 | 0.068897 | 0.89585 | 0.43841 | 0.17375 |
| 65 | 0.48093 | 0.22661 | 0.3134 | 0.80789 | 0.64066 | 0.54541 | 0.72796 | 1.0423 | 0.4217 |
| 66 | 0.58174 | 1.8116 | 0.079876 | 1.0338 | 1.5788 | 1.0223 | 1.4149 | 1.6423 | 0.44724 |
| 67 | 0.49885 | 1.5516 | 0.19493 | 1.1199 | 1.6026 | 1.5362 | 1.0699 | 1.7936 | 0.44101 |
| 68 | 0.50945 | 1.4452 | 0.68206 | 0.69244 | 1.1517 | 0.60686 | 1.1006 | 0.21487 | 0.38332 |
| 69 | 0.56831 | 0.73499 | 1.7534 | 1.153 | 1.9073 | 0.60472 | 1.2043 | 0.11578 | 1.0719 |
| 70 | 0.35045 | 1.6392 | 1.9315 | 1.3757 | 1.608 | 0.84073 | 1.1991 | 0.32045 | 0.040522 |
| 71 | 0.77096 | 0.23039 | 1.9024 | 0.8374 | 0.90484 | 0.76728 | 0.66205 | 0.13866 | 0.72413 |
| 72 | 0.61872 | 0.91911 | 1.4064 | 0.76576 | 1.8024 | 0.40487 | 1.0846 | 0.35915 | 1.1351 |
| 73 | 1.3514 | 1.6168 | 1.1378 | 1.0156 | 1.0752 | 1.3952 | 1.4347 | 0.063783 | 0.19978 |
| 74 | 0.92069 | 1.0245 | 1.9659 | 0.97477 | 0.37778 | 1.8194 | 0.81004 | 0.049457 | 0.17789 |
| 75 | 1.2334 | 1.7877 | 0.21762 | 1.2504 | 0.32474 | 0.27751 | 1.0202 | 0.061794 | 0.44185 |
| 76 | 0.75832 | 1.2503 | 1.1136 | 1.0429 | 0.13121 | 1.4917 | 1.4983 | 0.22185 | 0.87995 |
| 77 | 0.95632 | 0.91835 | 1.123 | 0.63738 | 1.7173 | 1.5591 | 1.0954 | 0.21773 | 0.05376 |
| 78 | 0.4009 | 1.0939 | 1.8172 | 1.1843 | 1.2446 | 1.1209 | 1.7757 | 1.0439 | 0.33293 |
| 79 | 0.6266 | 0.4459 | 0.19699 | 0.88236 | 0.3144 | 1.088 | 1.3922 | 0.46849 | 0.33305 |
| 80 | 0.44755 | 0.92691 | 1.3046 | 0.9558 | 1.247 | 0.3494 | 0.87107 | 0.22738 | 0.50015 |
| 81 | 1.3355 | 0.39713 | 1.0275 | 0.85927 | 0.24002 | 1.4535 | 0.96986 | 0.045963 | 1.2975 |
| 82 | 0.47707 | 0.13234 | 0.84244 | 1.2079 | 1.0687 | 1.6716 | 1.6291 | 0.43813 | 0.1109 |
| 83 | 0.3601 | 1.4458 | 0.21092 | 0.46238 | 0.47678 | 1.1315 | 1.5593 | 1.039 | 0.032401 |
| 84 | 1.1775 | 0.98598 | 0.9538 | 1.0315 | 1.2507 | 1.9644 | 1.3903 | 0.085612 | 0.57877 |
| 85 | 0.37365 | 1.4568 | 1.6749 | 1.35 | 0.051758 | 0.46017 | 0.3069 | 0.18629 | 0.29419 |
| 86 | 0.56189 | 0.54048 | 1.5077 | 0.97568 | 0.11061 | 0.12272 | 1.4994 | 0.82422 | 0.72368 |
| 87 | 0.62993 | 1.6795 | 1.1092 | 0.8493 | 0.31093 | 1.1489 | 1.6588 | 1.1155 | 0.85555 |
| 88 | 0.58918 | 1.2465 | 1.7601 | 1.0568 | 1.184 | 0.62697 | 1.1229 | 1.8256 | 0.19364 |
| 89 | 0.74942 | 0.60843 | 0.84965 | 1.0093 | 0.84317 | 0.64564 | 1.7941 | 0.14793 | 0.58356 |
| 90 | 0.41198 | 1.0625 | 1.416 | 0.73458 | 1.5144 | 1.2185 | 0.91422 | 1.4014 | 0.19331 |
| 91 | 0.41352 | 0.37393 | 1.8897 | 1.8725 | 0.10119 | 0.75585 | 1.4666 | 0.93118 | 0.02415 |
| 92 | 0.81322 | 0.86265 | 1.0827 | 0.82703 | 0.33125 | 0.54048 | 1.0167 | 0.24847 | 0.010357 |
| 93 | 0.24205 | 0.51455 | 0.6264 | 1.2796 | 0.86488 | 1.6751 | 0.71053 | 0.96754 | 0.029678 |
| 94 | 0.51855 | 1.18 | 0.84209 | 1.5874 | 0.86034 | 0.75204 | 1.8619 | 0.26973 | 1.6699 |
| 95 | 1.067 | 0.88381 | 1.8571 | 1.4996 | 1.91 | 0.36935 | 0.58401 | 0.10847 | 1.4285 |
| 96 | 0.44443 | 0.11414 | 1.2143 | 1.1745 | 0.085938 | 0.6933 | 1.879 | 1.1945 | 0.48424 |
| 97 | 0.74503 | 1.5998 | 1.7277 | 1.0811 | 1.2907 | 0.23129 | 1.48 | 0.030048 | 0.86394 |
| 98 | 0.98993 | 0.83914 | 1.8753 | 0.53079 | 1.2582 | 1.2079 | 0.80946 | 0.13146 | 0.85116 |
| 99 | 0.56222 | 0.50966 | 0.80294 | 0.37367 | 0.72531 | 1.2406 | 1.2009 | 1.4703 | 0.057764 |
| 100 | 0.45445 | 1.4985 | 0.64622 | 0.55191 | 1.84 | 1.5941 | 0.010824 | 1.6382 | 1.8306 |
| 101 | 0.30922 | 1.9335 | 0.066029 | 1.6134 | 1.7151 | 0.49428 | 1.2377 | 1.8353 | 0.052276 |
| 102 | 0.63477 | 0.9034 | 0.90135 | 0.55782 | 0.012051 | 1.1838 | 1.791 | 1.467 | 0.27511 |
| 103 | 0.47461 | 0.49938 | 0.040702 | 1.0518 | 0.32511 | 0.84021 | 0.54974 | 1.7907 | 0.95478 |
| 104 | 0.81873 | 1.6896 | 0.71319 | 1.0911 | 0.87918 | 1.724 | 0.82313 | 0.2665 | 0.9429 |
| 105 | 0.62027 | 1.3622 | 0.62802 | 0.68153 | 0.83244 | 1.609 | 0.88129 | 0.73149 | 0.88609 |
| 106 | 0.49577 | 1.6725 | 1.7879 | 1.1065 | 0.10621 | 1.681 | 0.97343 | 0.49689 | 0.26776 |
| 107 | 1.0342 | 1.1039 | 0.46689 | 0.79774 | 0.69913 | 1.7208 | 0.82967 | 0.051006 | 0.83507 |
| 108 | 0.36249 | 0.44734 | 0.94152 | 1.8266 | 0.43092 | 0.41989 | 1.309 | 1.5463 | 0.20857 |
| 109 | 0.44845 | 0.28156 | 0.73707 | 0.73044 | 1.1551 | 0.37714 | 0.57356 | 1.5452 | 0.86016 |
| 110 | 0.51272 | 0.11178 | 0.018055 | 0.42709 | 1.9318 | 1.0866 | 1.0406 | 1.2885 | 0.35132 |
| 111 | 0.54416 | 1.9431 | 1.9205 | 0.70343 | 1.3885 | 1.2182 | 0.043245 | 0.89989 | 1.2665 |
| 112 | 0.98235 | 1.2943 | 0.35899 | 0.55449 | 0.082508 | 1.35 | 1.5777 | 0.12237 | 0.066811 |
| 113 | 0.57801 | 1.071 | 1.2761 | 1.4613 | 0.28821 | 0.69381 | 1.1554 | 1.6904 | 0.37512 |
| 114 | 0.44592 | 1.1051 | 1.8896 | 0.54387 | 0.31283 | 1.3549 | 1.1225 | 0.93125 | 0.058118 |
| 115 | 0.52212 | 0.90302 | 1.8697 | 0.97063 | 1.8902 | 0.33894 | 0.87692 | 1.193 | 0.52877 |
| 116 | 0.54685 | 0.90559 | 1.0921 | 1.13 | 0.3968 | 1.123 | 1.5968 | 0.52962 | 1.0452 |
| 117 | 0.70059 | 1.6393 | 1.7323 | 1.3904 | 0.76396 | 1.183 | 1.5892 | 0.057335 | 0.90707 |
| 118 | 0.72114 | 1.9172 | 1.4457 | 0.98196 | 1.8933 | 1.4509 | 1.1716 | 0.26429 | 0.02776 |
| 119 | 0.57866 | 0.64344 | 0.43591 | 1.1735 | 1.1274 | 0.47057 | 1.5332 | 0.16761 | 0.93177 |
| 120 | 0.59387 | 1.4609 | 0.55244 | 1.003 | 0.2685 | 0.41076 | 1.0534 | 0.61535 | 0.31044 |
| 121 | 1.5576 | 0.50388 | 0.75733 | 0.7799 | 0.73194 | 1.6643 | 1.8433 | 0.041245 | 1.7743 |
| 122 | 0.54328 | 0.73981 | 0.5481 | 0.78774 | 0.71807 | 1.8149 | 1.5238 | 1.9653 | 0.49145 |
| 123 | 0.67183 | 0.07546 | 1.9794 | 1.1995 | 1.4205 | 0.56063 | 1.5468 | 0.20743 | 0.45724 |
| 124 | 0.80205 | 1.4118 | 0.26109 | 1.1839 | 0.94115 | 1.629 | 1.4735 | 0.20879 | 0.45584 |
| 125 | 0.43767 | 1.4697 | 1.9971 | 1.1178 | 1.9574 | 0.28368 | 0.25011 | 0.30081 | 0.43821 |
| 126 | 0.67045 | 0.68876 | 1.3308 | 0.46189 | 1.5727 | 1.8627 | 1.8686 | 0.31591 | 1.5787 |
| 127 | 0.62166 | 1.4654 | 1.5302 | 1.3938 | 1.1241 | 0.94266 | 1.7469 | 1.825 | 0.28877 |
| 128 | 0.56625 | 1.811 | 1.3591 | 1.7845 | 1.358 | 0.048269 | 0.65604 | 1.3515 | 0.69714 |
| 129 | 1.6599 | 1.3257 | 0.62373 | 0.76636 | 0.44642 | 1.2096 | 1.1107 | 0.011022 | 0.1705 |
| 130 | 0.30663 | 0.56992 | 1.013 | 1.574 | 1.3024 | 1.3326 | 0.41857 | 1.4782 | 0.17976 |
| 131 | 0.63695 | 1.8955 | 1.2059 | 0.68859 | 1.6957 | 1.3047 | 1.4644 | 1.5975 | 0.071114 |
| 132 | 0.40015 | 1.383 | 1.4544 | 1.3323 | 0.5403 | 0.1839 | 0.55301 | 0.92123 | 0.021093 |
| 133 | 1.9988 | 0.44514 | 1.4893 | 1.9997 | 0.13036 | 0.26997 | 1.6467 | 0.01958 | 1.3617 |
| 134 | 0.68675 | 0.11731 | 1.6866 | 0.5836 | 1.0304 | 0.37499 | 1.9464 | 1.0096 | 0.93498 |
| 135 | 1.0265 | 1.458 | 1.0017 | 1.7196 | 1.1587 | 0.3392 | 1.5914 | 0.14574 | 1.192 |
| 136 | 0.38839 | 1.8064 | 1.5188 | 0.74516 | 1.5828 | 0.24142 | 0.39755 | 0.38021 | 0.14689 |
| 137 | 0.38529 | 1.393 | 1.5225 | 1.3864 | 1.2426 | 1.3951 | 1.0388 | 1.002 | 0.059089 |
| 138 | 0.37995 | 1.4263 | 1.6836 | 1.5379 | 0.23712 | 0.88206 | 0.47032 | 0.037299 | 0.20002 |
| 139 | 0.62005 | 1.3668 | 1.0125 | 0.50141 | 0.42211 | 1.1354 | 1.6112 | 0.48106 | 0.87422 |
| 140 | 0.20802 | 0.66964 | 0.81691 | 1.9967 | 0.17295 | 1.6044 | 0.2395 | 1.1108 | 0.041457 |
| 141 | 0.27422 | 0.50748 | 0.39275 | 1.658 | 1.0042 | 0.24533 | 0.41721 | 1.7798 | 0.10745 |
| 142 | 0.70658 | 0.70387 | 1.1622 | 0.97997 | 1.3259 | 0.29235 | 1.9531 | 0.13296 | 0.23254 |
| 143 | 0.40454 | 0.88217 | 1.4045 | 1.2829 | 0.083192 | 0.76008 | 0.18857 | 1.3745 | 0.25251 |
| 144 | 0.3624 | 0.85525 | 0.40366 | 1.2915 | 0.16798 | 1.3433 | 0.30741 | 1.2925 | 0.10432 |
| 145 | 0.60163 | 0.89448 | 1.657 | 1.1365 | 1.8772 | 0.31605 | 1.2263 | 1.2678 | 1.2786 |
| 146 | 0.83008 | 0.057923 | 1.4575 | 1.4472 | 0.31888 | 0.2725 | 1.9692 | 0.19499 | 1.8927 |
| 147 | 0.66373 | 1.2532 | 1.6858 | 1.0135 | 0.88786 | 1.3705 | 1.9697 | 0.055586 | 0.32558 |
| 148 | 0.62318 | 1.3095 | 0.089823 | 1.3567 | 0.27149 | 0.30729 | 0.40495 | 0.36872 | 0.39539 |
| 149 | 0.48951 | 1.0004 | 1.4876 | 0.40139 | 1.3128 | 1.4775 | 1.4467 | 0.5927 | 0.067268 |
| 150 | 0.37449 | 0.54181 | 1.5774 | 1.0092 | 1.4446 | 0.63545 | 0.99725 | 1.9398 | 0.34322 |
| 151 | 0.31679 | 1.378 | 1.5607 | 1.6816 | 1.5473 | 1.3333 | 1.295 | 1.5851 | 0.15545 |
| 152 | 0.37837 | 1.3065 | 0.3491 | 0.75552 | 1.377 | 0.37319 | 0.034062 | 0.073496 | 0.18062 |
| 153 | 0.48769 | 0.19128 | 0.2027 | 1.0084 | 0.18072 | 0.24042 | 1.9918 | 1.2638 | 0.015011 |
| 154 | 0.54009 | 0.46401 | 1.149 | 1.4909 | 0.78187 | 0.57865 | 1.8172 | 0.24167 | 0.37012 |
| 155 | 0.77227 | 0.86718 | 1.9988 | 0.93535 | 1.8966 | 0.8549 | 1.4359 | 0.3549 | 1.4193 |
| 156 | 0.41492 | 0.92403 | 1.0767 | 1.4285 | 0.92852 | 0.13432 | 0.39628 | 1.891 | 0.59374 |
| 157 | 0.48204 | 0.5366 | 1.7803 | 1.4184 | 0.52027 | 0.42076 | 1.5026 | 1.347 | 0.3698 |
| 158 | 0.65348 | 1.8883 | 1.6629 | 1.5434 | 1.6077 | 0.66799 | 1.773 | 0.91816 | 0.35573 |
| 159 | 0.81858 | 0.66625 | 1.5152 | 0.69535 | 1.5321 | 0.80736 | 0.48388 | 0.031705 | 0.22913 |
| 160 | 0.63227 | 1.4987 | 0.09627 | 0.91122 | 1.6636 | 0.91045 | 1.191 | 0.47886 | 0.11834 |
| 161 | 0.52268 | 0.89397 | 0.27999 | 1.0121 | 0.59372 | 1.2107 | 1.2428 | 1.089 | 0.82762 |
| 162 | 0.61063 | 0.4697 | 1.8716 | 0.70023 | 0.10948 | 1.0561 | 1.727 | 0.69563 | 0.009072 |
| 163 | 0.58205 | 0.36258 | 1.4978 | 0.37325 | 0.57956 | 1.3517 | 1.4367 | 1.3096 | 0.80543 |
| 164 | 1.5926 | 1.5812 | 0.18623 | 1.0635 | 0.33755 | 0.48125 | 0.56223 | 0.016129 | 0.72653 |
| 165 | 0.52256 | 1.1958 | 0.29193 | 1.2559 | 1.1793 | 0.21414 | 0.84651 | 0.76657 | 0.34103 |
| 166 | 1.4897 | 1.9191 | 0.041415 | 1.7174 | 1.1961 | 0.58631 | 0.30973 | 0.005767 | 1.8586 |
| 167 | 0.84597 | 0.70977 | 1.0717 | 0.6059 | 1.4203 | 1.3431 | 1.7455 | 0.21971 | 1.1242 |
| 168 | 0.5405 | 1.6789 | 1.4933 | 1.0372 | 1.9791 | 1.6432 | 1.3738 | 0.6065 | 0.044328 |
| 169 | 0.91475 | 1.7682 | 0.32693 | 1.3972 | 0.65572 | 0.77239 | 0.54091 | 0.057461 | 1.5213 |
| 170 | 0.4519 | 0.44037 | 1.8699 | 1.1798 | 1.3164 | 1.6763 | 0.4527 | 1.5115 | 0.015646 |
| 171 | 0.88809 | 1.9966 | 1.2822 | 1.0279 | 0.37828 | 1.7252 | 0.92367 | 0.46184 | 0.096063 |
| 172 | 1.3471 | 1.2242 | 1.7078 | 1.1049 | 0.011945 | 1.9492 | 0.99115 | 0.057069 | 0.96081 |
| 173 | 0.517 | 1.3705 | 1.4626 | 1.5929 | 0.68732 | 0.39817 | 0.53926 | 1.1606 | 0.49098 |
| 174 | 1.2498 | 1.3777 | 1.0089 | 0.95666 | 0.59664 | 1.3219 | 0.077612 | 0.016424 | 0.41999 |
| 175 | 0.64563 | 0.048461 | 1.9514 | 0.41106 | 1.4242 | 1.8701 | 1.3086 | 1.0912 | 0.004455 |
| 176 | 0.52506 | 0.13613 | 1.4603 | 1.2908 | 1.1142 | 0.16658 | 1.3066 | 1.6917 | 1.6804 |
| 177 | 0.44026 | 1.0131 | 1.1157 | 1.3492 | 1.2569 | 0.19468 | 0.02039 | 1.7394 | 0.38783 |
| 178 | 0.25655 | 1.6384 | 0.6835 | 1.9455 | 1.7273 | 1.7176 | 1.919 | 0.21945 | 0.004944 |
| 179 | 0.67762 | 1.7181 | 0.75621 | 0.72089 | 1.7977 | 0.52737 | 0.66144 | 0.89538 | 0.47696 |
| 180 | 0.44093 | 0.22137 | 0.85914 | 1.2971 | 1.7854 | 0.26811 | 0.80113 | 1.2196 | 1.1057 |
| 181 | 0.59295 | 0.39889 | 0.003496 | 0.49977 | 0.69274 | 1.5045 | 1.383 | 1.5193 | 0.30674 |
| 182 | 0.28127 | 0.011777 | 1.3157 | 0.13207 | 1.9964 | 1.3763 | 1.5453 | 1.4568 | 0.089722 |
| 183 | 0.42423 | 1.7066 | 0.19937 | 0.39842 | 0.69798 | 1.3812 | 1.2436 | 1.056 | 0.097862 |
| 184 | 0.64289 | 1.4523 | 1.9468 | 1.7595 | 1.6249 | 0.06322 | 0.39206 | 1.0751 | 0.97483 |
| 185 | 0.61323 | 1.3034 | 0.56858 | 0.50911 | 1.9512 | 0.96613 | 1.5074 | 0.34982 | 0.52575 |
| 186 | 0.91248 | 0.37264 | 1.8811 | 1.1215 | 0.78539 | 0.074058 | 0.5878 | 0.1242 | 1.2977 |
| 187 | 0.59991 | 0.039094 | 1.9607 | 0.48564 | 0.40163 | 1.8404 | 1.589 | 1.2789 | 0.58779 |
| 188 | 0.75373 | 0.14471 | 0.93562 | 0.90072 | 0.71336 | 1.4064 | 1.4233 | 0.031313 | 0.89698 |
| 189 | 0.37549 | 1.9985 | 0.18734 | 0.33747 | 1.8649 | 1.0985 | 0.17468 | 1.3489 | 0.002561 |
| 190 | 0.38063 | 0.37884 | 1.4583 | 0.83297 | 0.10231 | 1.0336 | 0.41207 | 1.718 | 0.40441 |
| 191 | 0.41799 | 1.5781 | 1.0411 | 1.3148 | 1.9261 | 1.1248 | 0.8874 | 1.5765 | 0.091414 |
| 192 | 0.50825 | 0.84794 | 1.7523 | 0.65094 | 1.7488 | 0.99895 | 0.56191 | 1.4008 | 0.11013 |
| 193 | 1.0976 | 0.82418 | 0.40771 | 0.76922 | 0.38579 | 0.518 | 1.4824 | 0.085267 | 0.058575 |
| 194 | 0.3162 | 1.3476 | 0.80488 | 0.76257 | 0.081991 | 0.86135 | 0.40014 | 1.5618 | 0.033069 |
| 195 | 0.97298 | 1.2749 | 1.7975 | 0.62489 | 0.84404 | 0.81932 | 0.53039 | 0.013696 | 1.0855 |
| 196 | 0.43096 | 1.0034 | 0.84713 | 1.0182 | 0.98472 | 0.55137 | 0.49034 | 0.68153 | 0.43848 |
| 197 | 1.923 | 1.6557 | 1.506 | 1.1266 | 0.12413 | 0.7472 | 0.85792 | 0.026057 | 1.4321 |
| 198 | 0.35391 | 1.1869 | 0.78733 | 1.2045 | 1.0555 | 1.4517 | 0.085747 | 1.961 | 0.12112 |
| 199 | 0.55582 | 1.4693 | 1.5101 | 0.88868 | 0.036317 | 0.31219 | 0.2288 | 1.0393 | 1.2844 |
| 200 | 0.66427 | 1.794 | 1.0872 | 1.2717 | 0.62006 | 0.10817 | 0.40833 | 0.5446 | 0.44 |
| 201 | 0.58891 | 1.9339 | 0.45628 | 0.86577 | 1.4924 | 0.34792 | 1.1697 | 0.3937 | 0.4887 |
| 202 | 0.60385 | 1.0712 | 0.27664 | 1.1921 | 1.8271 | 0.92564 | 1.9352 | 1.0262 | 0.61569 |
| 203 | 0.67717 | 0.52124 | 1.1463 | 1.3764 | 1.8836 | 0.59537 | 1.8804 | 0.63815 | 0.98786 |
| 204 | 0.50325 | 0.63234 | 0.26865 | 0.92152 | 0.84764 | 0.70304 | 0.54131 | 0.94051 | 0.16884 |
| 205 | 0.32673 | 0.025874 | 1.9662 | 0.92538 | 0.075372 | 1.0083 | 1.6455 | 0.91746 | 0.024511 |
| 206 | 0.77646 | 1.6719 | 1.2148 | 0.54777 | 1.3557 | 1.247 | 0.30867 | 0.16678 | 1.022 |
| 207 | 0.65784 | 1.0073 | 1.1031 | 1.4224 | 0.5183 | 0.48788 | 1.1169 | 0.16609 | 0.25772 |
| 208 | 0.36339 | 1.5799 | 1.0209 | 0.96748 | 1.4941 | 1.2604 | 0.053345 | 1.3162 | 0.73727 |
| 209 | 0.49068 | 1.2816 | 0.93409 | 0.85582 | 1.6755 | 0.51037 | 1.9585 | 1.1631 | 0.040904 |
| 210 | 0.36313 | 1.4458 | 0.82912 | 1.7659 | 1.1829 | 0.81154 | 1.3839 | 0.28431 | 0.054704 |
| 211 | 0.4716 | 1.3074 | 1.2345 | 1.0389 | 1.0471 | 1.2566 | 1.2635 | 0.80088 | 0.3181 |
| 212 | 0.48343 | 1.5568 | 1.9537 | 1.2832 | 0.27541 | 0.23261 | 0.10824 | 0.97109 | 1.4689 |
| 213 | 0.49046 | 0.42816 | 1.7158 | 1.0843 | 1.8116 | 1.1142 | 0.062933 | 0.45255 | 1.2705 |
| 214 | 0.79565 | 1.4013 | 0.71705 | 0.99291 | 0.77213 | 1.9109 | 1.3367 | 0.37744 | 0.25158 |
| 215 | 0.52648 | 1.6786 | 1.7629 | 0.83462 | 1.8942 | 0.78429 | 0.25726 | 1.2779 | 0.15696 |
| 216 | 0.30482 | 1.4884 | 1.8921 | 0.70961 | 0.1804 | 1.6295 | 0.006092 | 0.51845 | 1.2347 |
| 217 | 0.42858 | 1.0518 | 1.1815 | 0.77066 | 1.5435 | 1.0382 | 1.0933 | 1.9959 | 0.39738 |
| 218 | 0.67016 | 1.3933 | 0.38508 | 0.35055 | 0.18768 | 1.7208 | 0.73646 | 0.63792 | 0.24 |
| 219 | 0.38118 | 0.42755 | 0.61118 | 1.4548 | 0.37551 | 0.79474 | 1.2307 | 1.2204 | 0.18901 |
| 220 | 0.50979 | 0.08626 | 0.77358 | 0.44782 | 0.40616 | 0.79923 | 1.4407 | 0.28365 | 0.34824 |
| 221 | 0.72695 | 0.12626 | 1.0464 | 0.87017 | 0.45514 | 1.5971 | 0.24573 | 0.21381 | 0.86799 |
| 222 | 0.72353 | 1.1165 | 0.55989 | 0.83598 | 0.29076 | 1.9894 | 0.16986 | 0.46928 | 0.070016 |
| 223 | 0.57581 | 0.71509 | 0.89918 | 1.0291 | 1.7063 | 0.21466 | 1.9828 | 1.518 | 0.9655 |
| 224 | 0.93634 | 1.3167 | 1.887 | 0.68562 | 1.253 | 0.65227 | 1.5139 | 0.35167 | 0.14648 |
| 225 | 0.43599 | 0.47337 | 0.85279 | 0.65976 | 1.8738 | 1.2152 | 1.0118 | 1.9535 | 0.21319 |
| 226 | 0.35847 | 0.18248 | 1.208 | 0.73475 | 1.3232 | 0.53557 | 0.2359 | 1.468 | 0.45274 |
| 227 | 0.79329 | 1.6721 | 0.45929 | 1.3228 | 0.49782 | 0.11121 | 1.3663 | 0.079197 | 0.18452 |
| 228 | 0.7117 | 0.1997 | 1.5025 | 0.75802 | 0.22665 | 0.24931 | 0.25869 | 0.42174 | 0.9616 |
| 229 | 0.27865 | 0.20967 | 1.9585 | 1.2612 | 1.8825 | 1.6373 | 0.8997 | 1.9058 | 0.054503 |
| 230 | 0.72377 | 1.2118 | 0.86606 | 0.61693 | 1.3788 | 1.2003 | 1.6776 | 0.11515 | 0.23429 |
| 231 | 0.8369 | 0.060633 | 1.7298 | 0.47235 | 0.92602 | 1.3392 | 0.38024 | 0.31375 | 0.94516 |
| 232 | 0.38324 | 0.88238 | 1.1657 | 0.8105 | 1.8666 | 1.9826 | 1.4994 | 1.1408 | 0.14526 |
| 233 | 0.74449 | 1.8321 | 0.95758 | 1.0397 | 1.7735 | 0.21062 | 1.8497 | 0.70026 | 0.10305 |
| 234 | 0.66342 | 0.81902 | 1.9613 | 0.80125 | 1.3265 | 1.9305 | 1.8709 | 1.9558 | 0.44609 |
| 235 | 0.76202 | 0.56383 | 1.284 | 1.0807 | 0.72686 | 0.65864 | 0.21674 | 0.054622 | 0.56167 |
| 236 | 0.77071 | 0.17644 | 1.3114 | 0.80989 | 1.7974 | 1.8113 | 1.9152 | 0.16052 | 0.60478 |
| 237 | 0.39041 | 1.6145 | 0.058442 | 1.3565 | 0.97277 | 0.10698 | 1.5687 | 1.5158 | 0.061991 |
| 238 | 0.60365 | 0.55329 | 0.9958 | 1.0652 | 0.74193 | 1.3954 | 1.2925 | 1.4658 | 0.33848 |
| 239 | 0.48996 | 1.2368 | 0.86154 | 1.1369 | 1.624 | 0.62048 | 0.080271 | 0.97123 | 1.1218 |
| 240 | 0.77129 | 1.3563 | 0.85807 | 1.0369 | 0.36997 | 1.418 | 0.56071 | 0.079742 | 0.054242 |
| 241 | 0.34255 | 0.91229 | 1.1716 | 0.87546 | 1.1827 | 0.38538 | 0.031883 | 1.7429 | 1.5646 |
| 242 | 0.40907 | 0.2611 | 1.6109 | 0.29705 | 0.067424 | 1.9218 | 0.89989 | 0.72948 | 0.23037 |
| 243 | 0.5416 | 0.51435 | 0.8678 | 1.0639 | 0.023226 | 0.90706 | 1.7088 | 0.0748 | 0.49739 |
| 244 | 0.66119 | 0.45475 | 1.6467 | 1.4458 | 0.19793 | 0.10704 | 0.51624 | 0.4225 | 0.43868 |
| 245 | 0.59178 | 0.4123 | 1.2132 | 1.0934 | 0.45556 | 0.41839 | 0.6828 | 1.0073 | 1.5677 |
| 246 | 0.8876 | 0.33054 | 1.0055 | 0.41572 | 1.162 | 1.749 | 1.7035 | 0.14504 | 0.32681 |
| 247 | 0.48072 | 0.10183 | 1.8354 | 0.95145 | 1.3758 | 0.89455 | 0.035107 | 1.4225 | 1.6644 |
| 248 | 0.63194 | 0.40859 | 0.10036 | 1.2125 | 1.0822 | 0.60676 | 1.4323 | 0.19473 | 0.30936 |
| 249 | 0.93756 | 0.57224 | 1.3687 | 0.71411 | 0.30426 | 1.3765 | 0.002036 | 0.34955 | 1.9204 |
| 250 | 1.0898 | 1.7032 | 1.7149 | 0.92658 | 0.096201 | 1.6781 | 0.6972 | 0.053168 | 0.77465 |
| 251 | 1.9354 | 0.31641 | 1.9622 | 0.85642 | 0.89397 | 0.44262 | 1.2333 | 0.023584 | 1.5774 |
| 252 | 0.78757 | 0.79128 | 1.8166 | 0.47679 | 0.023053 | 0.83355 | 1.4099 | 0.32078 | 0.07157 |
| 253 | 0.31991 | 1.0489 | 1.8747 | 1.378 | 1.9728 | 1.2013 | 0.39441 | 1.8306 | 0.099797 |
| 254 | 0.59811 | 1.2125 | 0.81368 | 0.91285 | 0.67344 | 1.1839 | 0.43308 | 1.599 | 0.03049 |
| 255 | 0.97234 | 1.2066 | 1.9085 | 1.0058 | 0.44825 | 0.12968 | 1.0843 | 0.068964 | 0.89503 |
| 256 | 0.51955 | 0.78248 | 0.61631 | 1.5246 | 0.071156 | 0.95402 | 1.9643 | 0.18653 | 1.7545 |
| 257 | 0.90537 | 1.2567 | 1.8104 | 0.77831 | 1.8651 | 0.96216 | 0.33258 | 0.13409 | 1.6488 |
| 258 | 0.57091 | 0.16557 | 0.80053 | 0.6616 | 0.86303 | 1.8153 | 0.89277 | 0.74417 | 0.5228 |
| 259 | 0.64344 | 1.1144 | 1.8006 | 0.70086 | 1.5901 | 1.6251 | 1.8527 | 1.3799 | 0.069093 |
| 260 | 0.61499 | 1.9303 | 0.49116 | 0.45426 | 1.8072 | 0.64591 | 1.056 | 0.22984 | 0.47825 |
| 261 | 1.1926 | 1.4004 | 1.3129 | 1.5041 | 0.79338 | 0.18862 | 0.19392 | 0.030501 | 1.7969 |
| 262 | 0.52681 | 1.3174 | 0.30924 | 0.88729 | 0.84113 | 1.2229 | 1.2993 | 1.4716 | 0.032967 |
| 263 | 0.26498 | 0.97358 | 0.36986 | 1.544 | 1.2135 | 1.6567 | 1.5661 | 0.19578 | 0.035149 |
| 264 | 0.63352 | 1.5813 | 1.7065 | 0.95986 | 1.8805 | 0.27014 | 0.19607 | 0.36888 | 1.1532 |
| 265 | 0.2341 | 1.1532 | 1.764 | 0.64393 | 0.85745 | 1.7016 | 0.04863 | 1.6611 | 0.039086 |
| 266 | 0.6131 | 1.6444 | 0.53353 | 1.0798 | 1.8772 | 1.1918 | 0.26115 | 0.05064 | 0.20218 |
| 267 | 0.65262 | 0.85748 | 0.7462 | 0.85292 | 0.2226 | 1.7285 | 1.5618 | 1.1508 | 0.49914 |
| 268 | 0.56351 | 0.5593 | 1.6423 | 0.69102 | 1.7433 | 1.5383 | 0.5271 | 1.0468 | 0.53567 |
| 269 | 0.50701 | 0.92131 | 0.86479 | 1.2035 | 0.46217 | 1.3008 | 0.65364 | 1.1811 | 0.037571 |
| 270 | 1.0532 | 1.7627 | 0.63838 | 1.0372 | 1.7572 | 1.5051 | 1.754 | 0.096501 | 0.36107 |
| 271 | 0.50874 | 1.7206 | 0.68886 | 0.47044 | 1.9157 | 1.8761 | 0.24512 | 1.8986 | 0.21433 |
| 272 | 0.52235 | 1.45 | 1.8359 | 0.71742 | 0.57205 | 1.6458 | 0.80625 | 1.3155 | 0.60529 |
| 273 | 0.345 | 0.56179 | 0.87294 | 0.87252 | 1.1167 | 1.0541 | 1.3871 | 0.74505 | 0.13073 |
| 274 | 0.45867 | 0.78112 | 1.5178 | 1.6066 | 0.46544 | 1.6128 | 1.4222 | 0.17771 | 0.087855 |
| 275 | 0.59019 | 0.19949 | 0.73138 | 0.60626 | 0.10761 | 0.69987 | 0.44232 | 0.98602 | 1.0757 |
| 276 | 0.8418 | 0.013286 | 1.595 | 0.57108 | 0.12838 | 0.82255 | 0.93367 | 0.42714 | 0.40828 |
| 277 | 0.73026 | 0.97396 | 0.75101 | 1.1046 | 0.20997 | 1.8724 | 1.7186 | 0.17588 | 0.55147 |
| 278 | 0.52291 | 1.9012 | 0.52394 | 1.0411 | 1.0434 | 0.62675 | 0.06231 | 1.2477 | 1.9693 |
| 279 | 0.69285 | 1.2882 | 1.2451 | 0.69958 | 0.20942 | 1.9677 | 0.44729 | 1.2873 | 0.25896 |
| 280 | 0.58659 | 1.4043 | 1.801 | 0.83245 | 1.142 | 1.6245 | 0.13958 | 0.7261 | 0.46775 |
| 281 | 0.54846 | 0.4232 | 1.1694 | 1.1626 | 1.6005 | 0.87908 | 0.76975 | 0.40995 | 0.065444 |
| 282 | 0.52744 | 0.56337 | 0.33836 | 0.7995 | 1.975 | 0.88175 | 1.4681 | 0.35852 | 0.68632 |
| 283 | 0.6486 | 1.0459 | 1.9519 | 0.67547 | 0.78503 | 1.5354 | 0.3264 | 0.68292 | 0.22961 |
| 284 | 0.83386 | 1.3453 | 1.3705 | 0.81982 | 0.62784 | 0.22353 | 1.0421 | 0.068055 | 0.19792 |
| 285 | 0.45741 | 1.1578 | 1.4311 | 1.2483 | 0.56643 | 1.1625 | 1.6215 | 1.5492 | 0.13513 |
| 286 | 0.46648 | 0.61693 | 0.75476 | 1.0002 | 0.66416 | 1.7471 | 1.5264 | 1.4451 | 0.20137 |
| 287 | 0.30458 | 1.2895 | 1.0871 | 1.9877 | 1.038 | 0.48375 | 1.253 | 1.879 | 0.17498 |
| 288 | 0.26418 | 0.39629 | 0.19823 | 0.87987 | 1.2524 | 1.8202 | 1.8638 | 1.399 | 0.050812 |
| 289 | 0.50166 | 1.9214 | 1.6782 | 1.9015 | 0.95456 | 0.68525 | 1.3431 | 1.57 | 0.19067 |
| 290 | 0.74004 | 0.29584 | 0.91677 | 0.50897 | 0.049004 | 1.9637 | 0.042446 | 0.21754 | 1.0951 |
| 291 | 0.56324 | 0.73357 | 1.6074 | 0.98923 | 1.3864 | 1.3215 | 1.6699 | 1.1333 | 1.6139 |
| 292 | 0.77194 | 1.7308 | 1.247 | 0.72928 | 0.95942 | 1.3397 | 0.15423 | 0.13069 | 0.092008 |
| 293 | 0.91337 | 1.6015 | 1.0448 | 1.3515 | 0.048625 | 0.21387 | 1.8659 | 0.21234 | 0.39095 |
| 294 | 0.87345 | 0.013012 | 1.7981 | 1.2026 | 1.9627 | 1.5222 | 1.936 | 0.001746 | 0.85665 |
| 295 | 0.88791 | 1.4415 | 1.7054 | 1.3704 | 1.9388 | 0.91221 | 1.3945 | 0.18068 | 0.48549 |
| 296 | 0.68286 | 1.4566 | 1.1411 | 0.93365 | 0.40204 | 0.7642 | 1.3217 | 1.0901 | 0.066572 |
| 297 | 0.58994 | 0.321 | 1.8589 | 1.4922 | 0.81432 | 0.18799 | 1.5809 | 1.6838 | 0.98045 |
| 298 | 0.77161 | 1.2168 | 0.006115 | 0.8841 | 1.0922 | 0.95544 | 1.9766 | 0.24762 | 0.77866 |
| 299 | 0.62437 | 1.4254 | 0.9763 | 1.0066 | 1.8664 | 0.6768 | 0.20218 | 0.2891 | 0.65531 |
| 300 | 0.60298 | 1.1287 | 0.059769 | 0.99502 | 0.39456 | 0.29087 | 0.31806 | 0.28479 | 0.37125 |
| 301 | 0.44141 | 1.163 | 1.7283 | 1.0051 | 1.3568 | 0.67334 | 0.67016 | 1.2223 | 0.20414 |
| 302 | 0.40611 | 0.77709 | 0.10041 | 0.65183 | 1.114 | 1.3261 | 0.88093 | 0.44049 | 0.037284 |
| 303 | 0.67884 | 1.2622 | 0.11789 | 1.101 | 0.41849 | 0.55633 | 0.88299 | 0.47219 | 0.37229 |
| 304 | 0.33447 | 0.7295 | 1.188 | 0.76488 | 0.5339 | 1.2758 | 0.016433 | 1.4883 | 1.9367 |
| 305 | 0.8124 | 0.34265 | 0.69103 | 1.2392 | 0.33336 | 1.2361 | 1.9516 | 0.047774 | 0.36691 |
| 306 | 0.73542 | 1.81 | 1.5112 | 0.59143 | 1.1995 | 0.52974 | 0.75161 | 0.5613 | 0.45054 |
| 307 | 0.38948 | 1.0518 | 0.79221 | 0.45792 | 1.9153 | 0.55271 | 1.9217 | 1.6759 | 0.09739 |
| 308 | 0.45037 | 1.887 | 0.89495 | 1.3106 | 1.7216 | 0.063468 | 0.76897 | 0.77559 | 0.15378 |
| 309 | 0.55876 | 1.4603 | 1.7092 | 1.2879 | 1.2058 | 0.3069 | 1.8012 | 0.033445 | 0.25004 |
| 310 | 0.58198 | 0.95289 | 1.6228 | 1.5256 | 1.6712 | 0.78267 | 0.0423 | 1.2662 | 0.6514 |
| 311 | 0.58284 | 1.8183 | 1.2727 | 1.6377 | 0.61758 | 0.3144 | 0.37529 | 0.21172 | 1.0169 |
| 312 | 1.0542 | 1.8328 | 1.8499 | 0.74046 | 1.7452 | 1.8122 | 1.4492 | 0.039031 | 0.72592 |
| 313 | 0.82973 | 0.70934 | 1.6736 | 0.93168 | 1.3526 | 0.33713 | 0.8249 | 0.26722 | 0.28016 |
| 314 | 0.80917 | 1.3806 | 0.24802 | 0.81174 | 0.24964 | 0.63704 | 1.3679 | 0.074475 | 0.044522 |
| 315 | 0.58452 | 1.7491 | 0.74296 | 0.9735 | 0.22846 | 0.42694 | 0.24459 | 1.9711 | 0.1548 |
| 316 | 0.74812 | 1.4455 | 0.68763 | 1.4436 | 1.2963 | 0.11003 | 1.1661 | 0.18033 | 0.91237 |
| 317 | 0.47622 | 1.2917 | 1.5251 | 0.5243 | 0.39985 | 0.69166 | 1.9807 | 1.5779 | 0.18147 |
| 318 | 0.63997 | 0.022945 | 0.78665 | 1.0867 | 0.17326 | 1.0019 | 0.89675 | 0.19818 | 1.3283 |
| 319 | 0.60276 | 0.62018 | 0.98274 | 0.88594 | 1.5228 | 0.35374 | 1.6689 | 0.36732 | 0.19145 |
| 320 | 0.68267 | 0.87529 | 1.0609 | 0.58641 | 0.12607 | 1.4788 | 1.3231 | 1.0915 | 0.51596 |
| 321 | 0.28955 | 1.6946 | 1.5394 | 0.58816 | 0.22043 | 0.86258 | 1.2131 | 1.9183 | 0.043226 |
| 322 | 0.74553 | 0.75919 | 0.090779 | 0.68771 | 0.58997 | 0.33211 | 0.1258 | 0.053587 | 0.029583 |
| 323 | 0.39507 | 0.85901 | 1.1473 | 1.5702 | 1.4488 | 0.23332 | 0.51808 | 1.8761 | 0.18044 |
| 324 | 0.72639 | 0.57622 | 1.3898 | 0.41759 | 1.9241 | 1.4667 | 1.554 | 0.3332 | 1.1659 |
| 325 | 0.73616 | 0.48639 | 0.71037 | 0.46907 | 1.4821 | 1.373 | 0.31277 | 0.43358 | 0.59614 |
| 326 | 0.37128 | 1.1201 | 1.365 | 1.1437 | 1.789 | 0.22152 | 0.002493 | 0.42218 | 0.92566 |
| 327 | 0.60326 | 1.153 | 0.23634 | 1.3389 | 1.8033 | 1.268 | 1.5304 | 0.097978 | 0.8422 |
| 328 | 0.68458 | 0.99472 | 1.0868 | 0.87793 | 1.1578 | 1.1811 | 1.0643 | 1.0347 | 0.38437 |
| 329 | 0.50493 | 0.2585 | 0.043875 | 1.7254 | 0.46675 | 0.16269 | 1.9131 | 0.001015 | 1.3348 |
| 330 | 0.82457 | 0.8707 | 1.4563 | 1.6503 | 0.79714 | 0.050307 | 1.288 | 0.19201 | 0.7026 |
| 331 | 0.65937 | 1.4099 | 1.7688 | 1.2822 | 0.71414 | 0.24801 | 0.004361 | 0.47039 | 1.5795 |
| 332 | 0.54597 | 0.68208 | 1.5097 | 1.0617 | 1.0278 | 1.5155 | 0.15896 | 0.37019 | 0.67976 |
| 333 | 0.53976 | 0.32426 | 0.20911 | 1.0416 | 0.41019 | 0.98073 | 1.2481 | 1.6297 | 0.31304 |
| 334 | 0.30228 | 1.0322 | 1.3171 | 1.8836 | 1.288 | 1.6051 | 1.7355 | 0.86364 | 0.080093 |
| 335 | 0.53542 | 0.52948 | 0.47404 | 0.60713 | 0.23575 | 0.77841 | 0.57901 | 1.7891 | 0.23788 |
| 336 | 1.0799 | 1.3743 | 1.9687 | 0.51328 | 1.7331 | 1.6467 | 1.3588 | 0.01318 | 0.1487 |
| 337 | 0.88837 | 0.738 | 0.84783 | 0.47911 | 1.2059 | 0.6166 | 1.9494 | 0.1797 | 0.1139 |
| 338 | 0.54024 | 0.3992 | 1.493 | 0.27646 | 0.90529 | 1.3169 | 0.49212 | 0.42842 | 0.13398 |
| 339 | 1.2901 | 0.67273 | 1.815 | 0.80731 | 0.52181 | 0.7519 | 0.57489 | 0.032596 | 1.3259 |
| 340 | 0.66878 | 1.8051 | 1.0423 | 1.2435 | 1.0101 | 0.99159 | 0.78562 | 1.4134 | 0.5965 |
| 341 | 0.45433 | 0.64547 | 1.017 | 0.26077 | 1.9395 | 1.4679 | 0.6687 | 1.3965 | 0.085697 |
| 342 | 0.60683 | 0.27027 | 0.64903 | 0.59052 | 1.5372 | 1.517 | 1.8246 | 0.80919 | 0.89078 |
| 343 | 0.82307 | 0.56228 | 1.8488 | 1.4991 | 1.664 | 0.25158 | 1.3835 | 0.25519 | 1.3433 |
| 344 | 0.45765 | 0.59432 | 1.2876 | 0.37589 | 0.030213 | 1.2366 | 0.2789 | 0.28 | 0.34147 |
| 345 | 0.70849 | 1.5816 | 1.5679 | 0.92698 | 0.54734 | 1.6566 | 1.8415 | 0.4691 | 1.536 |
| 346 | 0.63803 | 0.63003 | 0.8506 | 1.8015 | 1.7343 | 0.032082 | 0.023252 | 0.043652 | 0.79281 |
| 347 | 0.29815 | 0.86352 | 1.4573 | 1.8136 | 1.5706 | 0.20023 | 0.025516 | 0.33234 | 0.90404 |
| 348 | 0.55426 | 0.23099 | 1.3481 | 0.43524 | 1.9063 | 0.51635 | 0.57172 | 1.6003 | 0.19893 |
| 349 | 0.72667 | 1.9799 | 1.8641 | 1.0284 | 0.73936 | 1.9284 | 0.16786 | 0.50552 | 1.2661 |
| 350 | 0.60316 | 0.076815 | 0.25445 | 0.94374 | 1.9352 | 0.30149 | 1.1253 | 1.42 | 0.15989 |
| 351 | 0.4982 | 0.37666 | 0.045543 | 0.31163 | 1.2729 | 0.97059 | 0.10154 | 0.91191 | 0.14355 |
| 352 | 0.62923 | 1.2776 | 1.9532 | 0.87811 | 1.3574 | 1.4966 | 0.10029 | 1.2266 | 0.77829 |
| 353 | 0.52341 | 1.8961 | 1.4483 | 0.72171 | 0.23922 | 0.39897 | 1.8339 | 0.87114 | 0.134 |
| 354 | 0.48705 | 1.5392 | 1.4887 | 0.72942 | 0.41687 | 0.8129 | 0.27941 | 0.30909 | 0.27057 |
| 355 | 0.4535 | 0.92434 | 1.4659 | 1.5758 | 1.0034 | 0.26191 | 0.046057 | 1.4117 | 0.27865 |
| 356 | 0.43801 | 1.0532 | 0.43202 | 1.7584 | 1.2775 | 0.10133 | 1.866 | 0.52266 | 1.3785 |
| 357 | 0.54892 | 0.20998 | 1.3009 | 1.0726 | 1.611 | 1.0533 | 1.8948 | 1.785 | 1.0242 |
| 358 | 0.66483 | 0.24272 | 0.008056 | 0.45546 | 1.7622 | 1.8179 | 0.57793 | 0.23542 | 0.7732 |
| 359 | 0.43978 | 0.45747 | 0.6436 | 1.2927 | 0.91359 | 1.9034 | 0.055217 | 0.020779 | 0.029902 |
| 360 | 0.6289 | 1.7261 | 1.929 | 0.5111 | 0.70302 | 1.8586 | 0.58528 | 0.93204 | 0.24976 |
| 361 | 0.54915 | 1.0195 | 1.77 | 1.7565 | 0.58159 | 0.16414 | 0.82693 | 1.1086 | 1.9303 |
| 362 | 0.53331 | 1.3146 | 0.84185 | 0.34834 | 0.042847 | 1.4102 | 0.40812 | 1.0578 | 0.26619 |
| 363 | 0.53094 | 0.59719 | 0.74486 | 0.79993 | 0.018637 | 1.1644 | 0.41964 | 1.7969 | 0.64212 |
| 364 | 0.84688 | 0.38747 | 0.2302 | 0.87772 | 0.99834 | 1.7188 | 1.2839 | 0.12124 | 1.6046 |
| 365 | 0.68818 | 1.3391 | 0.18185 | 0.82831 | 1.3998 | 1.3895 | 1.1223 | 0.84192 | 0.33673 |
| 366 | 0.6375 | 1.0249 | 1.9417 | 0.8459 | 0.65174 | 1.6619 | 1.5462 | 1.3974 | 0.41909 |
| 367 | 0.56055 | 1.5332 | 1.7355 | 1.1026 | 0.38445 | 0.13662 | 0.64844 | 1.0319 | 1.281 |
| 368 | 0.2833 | 1.0049 | 1.527 | 1.9062 | 1.2095 | 0.25937 | 0.11838 | 1.6883 | 0.2971 |
| 369 | 0.21552 | 0.37715 | 1.0009 | 0.71035 | 1.1882 | 0.53173 | 1.4969 | 1.8747 | 0.055947 |
| 370 | 0.38373 | 1.2853 | 1.5419 | 0.7836 | 1.9709 | 1.896 | 1.4638 | 1.6449 | 0.23906 |
| 371 | 0.56277 | 0.48916 | 1.1222 | 0.96879 | 0.2236 | 1.3687 | 1.0675 | 1.5761 | 1.1247 |
| 372 | 0.80341 | 1.615 | 1.5969 | 0.70718 | 0.97888 | 1.2135 | 1.434 | 0.18154 | 0.22506 |
| 373 | 0.84325 | 1.082 | 1.8125 | 1.0102 | 1.2351 | 0.70326 | 1.4288 | 0.19443 | 1.9633 |
| 374 | 0.55662 | 1.1334 | 0.23794 | 1.2457 | 1.7826 | 0.092888 | 0.58119 | 0.86872 | 0.30224 |
| 375 | 0.62676 | 1.734 | 0.76447 | 1.2731 | 1.5291 | 1.0034 | 1.6287 | 0.3174 | 1.9793 |
| 376 | 0.79684 | 1.5107 | 0.48831 | 1.4041 | 1.6886 | 0.64714 | 0.56537 | 0.16575 | 0.83325 |
| 377 | 0.99005 | 1.2388 | 1.5234 | 1.162 | 0.11343 | 1.7758 | 0.59079 | 0.041406 | 0.29547 |
| 378 | 0.39944 | 0.18503 | 1.8792 | 1.4398 | 0.80141 | 1.0129 | 0.99648 | 0.3461 | 0.37402 |
| 379 | 0.49018 | 0.96178 | 1.799 | 0.84966 | 0.31767 | 0.27902 | 0.52765 | 1.6343 | 0.37914 |
| 380 | 0.60927 | 0.84582 | 0.3058 | 0.70235 | 1.3448 | 1.2574 | 1.9805 | 1.333 | 0.27138 |
| 381 | 0.49327 | 0.64246 | 0.68107 | 0.74853 | 0.39328 | 1.0995 | 0.093218 | 1.5857 | 0.90739 |
| 382 | 0.6592 | 0.041588 | 0.87954 | 0.44061 | 1.4539 | 0.86106 | 0.80717 | 0.3442 | 1.0551 |
| 383 | 0.3288 | 1.5113 | 0.9598 | 0.67663 | 1.9924 | 0.27692 | 0.27603 | 1.4987 | 0.15825 |
| 384 | 0.62474 | 0.47041 | 0.49526 | 1.4623 | 0.58195 | 0.26018 | 1.5728 | 0.35134 | 1.483 |
| 385 | 0.59511 | 1.7441 | 1.2954 | 0.77383 | 1.9569 | 0.95042 | 0.87186 | 0.41446 | 0.70805 |
| 386 | 0.4703 | 0.40226 | 1.8398 | 0.27869 | 1.8318 | 1.3079 | 0.032648 | 1.0557 | 0.35976 |
| 387 | 0.47244 | 0.61159 | 0.86914 | 0.50359 | 1.6315 | 1.3563 | 0.15711 | 1.0026 | 0.17924 |
| 388 | 0.66706 | 1.9895 | 1.1561 | 1.569 | 0.28768 | 0.80222 | 1.3783 | 0.090708 | 0.1516 |
| 389 | 0.46281 | 0.29197 | 1.2901 | 1.2418 | 0.67561 | 1.4103 | 1.085 | 0.49663 | 0.03063 |
| 390 | 1.0394 | 0.88327 | 1.2841 | 1.0805 | 0.62118 | 1.0064 | 1.1994 | 0.17684 | 0.58426 |
| 391 | 0.84519 | 1.7577 | 1.3036 | 1.8041 | 0.17543 | 0.34117 | 1.5369 | 8.77E-05 | 1.1429 |
| 392 | 0.70774 | 1.9057 | 1.6698 | 1.2176 | 0.81342 | 0.82177 | 0.88672 | 0.3952 | 1.4767 |
| 393 | 0.50083 | 1.6896 | 1.2038 | 0.6615 | 1.1609 | 1.7347 | 1.1859 | 1.6459 | 0.42732 |
| 394 | 0.63679 | 0.079051 | 0.85225 | 0.30234 | 0.65558 | 1.2256 | 0.8865 | 1.1249 | 0.22753 |
| 395 | 0.71678 | 0.40286 | 1.7043 | 0.58893 | 1.835 | 1.1206 | 1.245 | 0.83589 | 0.71808 |
| 396 | 0.28071 | 0.74191 | 1.2043 | 0.41788 | 1.5484 | 1.5597 | 0.000721 | 1.6117 | 1.4982 |
| 397 | 0.51962 | 0.17766 | 0.94347 | 0.60084 | 0.26381 | 1.7858 | 1.6044 | 1.7264 | 0.6147 |
| 398 | 0.61523 | 0.7055 | 1.4057 | 0.57373 | 0.78455 | 0.60164 | 1.9018 | 0.87892 | 0.60698 |
| 399 | 0.37741 | 1.6976 | 0.30823 | 1.3416 | 0.83058 | 0.20293 | 1.4433 | 1.1583 | 0.08274 |
| 400 | 0.70418 | 1.4069 | 1.563 | 1.1811 | 0.27302 | 1.0014 | 0.97096 | 1.3992 | 0.75959 |
| 401 | 0.4606 | 1.6421 | 0.33727 | 1.2406 | 0.96704 | 0.081875 | 0.74664 | 0.99144 | 0.77958 |
| 402 | 0.45935 | 1.6175 | 0.59662 | 1.3077 | 0.2313 | 1.2754 | 1.9056 | 0.015032 | 0.89433 |
| 403 | 0.6675 | 1.9797 | 1.7128 | 0.71848 | 0.96596 | 1.5061 | 1.2446 | 1.0647 | 0.77022 |
| 404 | 0.50405 | 0.22145 | 0.13702 | 0.35569 | 0.39034 | 1.8019 | 0.54393 | 0.40497 | 0.22126 |
| 405 | 1.1614 | 0.40073 | 1.1907 | 1.1224 | 1.9846 | 0.81709 | 1.1217 | 0.060565 | 0.28928 |
| 406 | 1.2224 | 0.8511 | 1.7326 | 0.71838 | 1.6418 | 0.87503 | 1.0841 | 0.10113 | 0.80281 |
| 407 | 0.6432 | 0.56031 | 1.8912 | 1.0151 | 1.5451 | 1.4718 | 1.8175 | 1.9606 | 0.57905 |
| 408 | 0.57765 | 0.33458 | 1.4298 | 1.5271 | 1.05 | 0.34891 | 1.2721 | 0.7293 | 1.5807 |
| 409 | 0.67516 | 1.4378 | 1.1759 | 0.94657 | 1.8746 | 0.034778 | 1.2092 | 1.1582 | 0.68051 |
| 410 | 0.46594 | 1.6095 | 0.158 | 0.71115 | 0.26311 | 0.26873 | 1.158 | 0.87286 | 0.089415 |
| 411 | 0.53501 | 1.6745 | 1.2223 | 1.7907 | 1.1532 | 0.1128 | 1.6852 | 0.1924 | 1.815 |
| 412 | 0.50068 | 0.80276 | 0.73379 | 0.4884 | 0.20342 | 0.69199 | 0.38275 | 0.39685 | 0.23643 |
| 413 | 0.73012 | 0.13148 | 1.428 | 0.34809 | 0.40469 | 1.9016 | 0.78519 | 0.69128 | 0.32182 |
| 414 | 0.32202 | 1.8265 | 0.085143 | 1.3032 | 1.9994 | 0.91808 | 0.01051 | 1.45 | 1.5199 |
| 415 | 1.0389 | 1.1684 | 1.1006 | 0.7154 | 1.4264 | 0.75738 | 0.64009 | 0.072557 | 1.2516 |
| 416 | 0.80663 | 1.9244 | 1.221 | 1.1681 | 0.80707 | 0.17774 | 0.87939 | 0.075104 | 0.11597 |
| 417 | 0.51475 | 0.29351 | 0.70219 | 1.019 | 1.8332 | 1.0764 | 1.8368 | 0.77375 | 1.3585 |
| 418 | 0.90942 | 1.5386 | 0.67736 | 0.89803 | 0.67763 | 0.44534 | 1.255 | 0.11631 | 0.93569 |
| 419 | 0.80334 | 0.76613 | 1.5396 | 0.43502 | 1.8569 | 0.87153 | 0.54692 | 0.45959 | 0.41053 |
| 420 | 0.68716 | 1.5225 | 1.6808 | 1.7356 | 0.64926 | 1.4286 | 1.6197 | 0.14528 | 0.080665 |
| 421 | 0.35944 | 0.76172 | 1.4681 | 1.4886 | 0.013021 | 0.42456 | 0.32492 | 1.9598 | 0.21016 |
| 422 | 1.2773 | 0.39173 | 0.22716 | 1.9261 | 0.15459 | 0.20927 | 1.5477 | 0.021672 | 1.7899 |
| 423 | 0.59608 | 1.8416 | 1.3034 | 0.83574 | 1.6082 | 0.70649 | 1.5829 | 0.008885 | 0.25589 |
| 424 | 0.59376 | 1.1618 | 1.5762 | 1.5151 | 0.93476 | 0.11167 | 1.3597 | 1.2933 | 0.17278 |
| 425 | 0.52402 | 0.91036 | 0.66275 | 0.64249 | 1.2159 | 0.83511 | 1.658 | 1.5282 | 0.3333 |
| 426 | 0.4418 | 0.21465 | 0.48689 | 0.72667 | 1.9762 | 1.0146 | 1.5614 | 1.1487 | 0.22021 |
| 427 | 0.63149 | 0.13899 | 0.096584 | 1.0165 | 1.6116 | 1.4763 | 1.5088 | 0.64556 | 0.75573 |
| 428 | 0.78194 | 1.3392 | 1.6221 | 1.4102 | 1.8083 | 0.57466 | 1.8005 | 0.98457 | 0.66839 |
| 429 | 0.4922 | 0.60558 | 0.22519 | 0.31365 | 1.8618 | 1.764 | 0.48811 | 1.5428 | 0.2254 |
| 430 | 0.49952 | 0.54505 | 1.2948 | 1.2934 | 0.99328 | 0.52885 | 1.4181 | 0.87501 | 0.41164 |
| 431 | 0.61625 | 0.26846 | 1.532 | 0.58945 | 1.3578 | 1.3574 | 0.62985 | 1.7603 | 0.20337 |
| 432 | 0.35273 | 1.3928 | 1.5299 | 1.8565 | 1.8069 | 1.6924 | 0.66936 | 0.095692 | 0.15136 |
| 433 | 0.7846 | 0.43319 | 1.332 | 0.82728 | 0.041117 | 1.8233 | 1.5349 | 0.90204 | 0.16339 |
| 434 | 0.62907 | 0.84602 | 1.3762 | 0.64166 | 1.8622 | 0.18732 | 1.4787 | 1.9037 | 0.4163 |
| 435 | 0.37269 | 0.22233 | 1.1536 | 1.0834 | 0.60815 | 0.3733 | 1.1853 | 0.47001 | 0.16267 |
| 436 | 0.76343 | 0.56607 | 1.315 | 0.64483 | 1.6472 | 1.0668 | 0.48188 | 0.27524 | 0.092874 |
| 437 | 0.23707 | 1.4723 | 0.3063 | 1.3915 | 0.86943 | 0.67084 | 0.099377 | 0.32638 | 0.017125 |
| 438 | 1.0704 | 1.3517 | 1.3614 | 0.5945 | 0.6161 | 0.98128 | 1.2321 | 0.068736 | 1.2335 |
| 439 | 0.46265 | 0.006887 | 1.8448 | 0.43065 | 0.61069 | 1.3373 | 0.042158 | 1.2111 | 1.3013 |
| 440 | 0.30711 | 1.3658 | 0.46878 | 1.9355 | 0.94402 | 0.098365 | 1.683 | 1.8716 | 0.17507 |
| 441 | 0.56106 | 1.0162 | 0.75886 | 1.4037 | 1.8224 | 1.3787 | 1.356 | 0.23656 | 0.12013 |
| 442 | 0.54958 | 0.74464 | 0.60208 | 0.95891 | 0.41174 | 1.9309 | 0.81753 | 0.66129 | 0.63166 |
| 443 | 0.3463 | 1.2823 | 1.6296 | 1.5723 | 1.9003 | 0.40833 | 0.12318 | 1.7075 | 0.11813 |
| 444 | 0.34289 | 0.10145 | 1.587 | 0.73945 | 0.77876 | 1.1218 | 0.10179 | 0.32314 | 0.016102 |
| 445 | 0.43162 | 1.3748 | 1.0567 | 0.1835 | 0.95785 | 1.7119 | 0.26835 | 1.602 | 0.081468 |
| 446 | 0.90915 | 0.34678 | 1.0523 | 0.63639 | 1.3011 | 1.5216 | 0.46992 | 0.048179 | 0.065721 |
| 447 | 0.6154 | 1.3611 | 0.25365 | 0.70278 | 1.2751 | 0.63771 | 1.799 | 0.57145 | 0.40188 |
| 448 | 0.54484 | 1.5828 | 0.46944 | 1.3691 | 1.9144 | 1.4125 | 1.463 | 0.067726 | 0.074076 |
| 449 | 0.60881 | 1.6803 | 0.59682 | 0.52991 | 0.80795 | 1.3985 | 0.76276 | 1.1816 | 0.43522 |
| 450 | 0.53106 | 1.7734 | 1.1587 | 0.56485 | 1.8315 | 1.8133 | 1.019 | 0.58126 | 0.1483 |
| 451 | 0.45729 | 1.2351 | 1.6044 | 1.8416 | 0.71287 | 0.73795 | 1.8845 | 0.082412 | 0.25807 |
| 452 | 0.60131 | 1.1406 | 0.4698 | 1.2652 | 1.0813 | 0.075186 | 0.15554 | 0.23443 | 0.55681 |
| 453 | 0.55187 | 1.9903 | 1.4435 | 1.5666 | 1.8853 | 0.39973 | 0.88401 | 0.58143 | 0.77973 |
| 454 | 0.47389 | 1.3195 | 1.456 | 0.93693 | 1.7876 | 1.0391 | 0.03447 | 0.69069 | 1.2737 |
| 455 | 0.55742 | 1.276 | 0.25522 | 0.88083 | 1.6653 | 0.52363 | 0.95782 | 1.7011 | 0.2345 |
| 456 | 0.50606 | 0.64806 | 1.1048 | 0.90648 | 1.6651 | 1.831 | 1.5795 | 1.4096 | 0.060715 |
| 457 | 0.99798 | 1.8293 | 1.0553 | 1.8274 | 1.7034 | 0.61555 | 1.1667 | 0.014356 | 1.1882 |
| 458 | 0.86446 | 1.5892 | 0.54514 | 1.2001 | 1.0777 | 0.78588 | 0.4716 | 0.091274 | 0.98552 |
| 459 | 0.5631 | 1.7133 | 0.29833 | 1.4306 | 0.77118 | 0.17919 | 0.90291 | 1.8692 | 0.38919 |
| 460 | 0.30314 | 1.4918 | 1.1938 | 1.0289 | 0.018962 | 1.2899 | 0.58486 | 1.983 | 0.046327 |
| 461 | 0.92589 | 0.059981 | 0.75069 | 1.1761 | 0.53053 | 0.75686 | 1.4472 | 0.0259 | 0.67797 |
| 462 | 0.69666 | 1.9601 | 1.4827 | 0.65462 | 1.894 | 0.38068 | 0.085076 | 0.55696 | 0.083059 |
| 463 | 0.6609 | 1.3238 | 0.38357 | 0.92757 | 0.33051 | 0.99124 | 1.9025 | 0.88627 | 0.68171 |
| 464 | 0.59151 | 0.82001 | 0.50958 | 1.4482 | 1.618 | 0.6599 | 1.9885 | 0.33667 | 1.6367 |
| 465 | 0.79038 | 1.5519 | 1.2722 | 1.0762 | 0.79733 | 1.6229 | 0.368 | 0.2559 | 1.223 |
| 466 | 0.63316 | 0.72672 | 0.038749 | 0.4134 | 1.3661 | 1.6182 | 0.36137 | 0.19798 | 0.44228 |
| 467 | 0.67827 | 1.4958 | 0.51419 | 0.28976 | 1.515 | 1.1846 | 0.7643 | 0.93488 | 0.22091 |
| 468 | 0.45073 | 1.4366 | 1.6727 | 1.2071 | 0.13434 | 1.36 | 1.1158 | 0.27396 | 0.16295 |
| 469 | 0.68954 | 0.25138 | 1.8301 | 0.65237 | 1.5332 | 1.8006 | 1.2261 | 0.48384 | 0.75851 |
| 470 | 0.62325 | 1.0231 | 1.7167 | 1.5625 | 0.090013 | 0.18246 | 0.31524 | 0.26123 | 1.1209 |
| 471 | 0.63206 | 0.66848 | 1.6871 | 0.86877 | 0.52816 | 1.2949 | 1.3797 | 0.56583 | 0.73918 |
| 472 | 0.40474 | 0.56876 | 1.9572 | 1.6069 | 1.1133 | 0.23438 | 0.19622 | 0.70719 | 0.7671 |
| 473 | 0.84966 | 0.94597 | 1.3034 | 0.46264 | 0.48175 | 1.6541 | 1.9657 | 0.13946 | 0.82626 |
| 474 | 0.70422 | 0.28505 | 0.2778 | 0.69412 | 1.499 | 0.25136 | 0.17052 | 0.18823 | 0.67726 |
| 475 | 0.53263 | 1.8159 | 1.5442 | 1.3118 | 1.9179 | 0.36665 | 1.0431 | 1.0861 | 1.3842 |
| 476 | 0.65866 | 1.3773 | 1.621 | 0.82416 | 1.6508 | 1.6842 | 1.2741 | 0.77353 | 1.1321 |
| 477 | 0.76129 | 1.6321 | 1.2989 | 1.57 | 1.6648 | 0.55056 | 0.91631 | 0.17558 | 1.1004 |
| 478 | 1.0563 | 1.0286 | 0.55106 | 0.81321 | 0.34854 | 0.15391 | 1.5442 | 0.083504 | 0.30524 |
| 479 | 0.57429 | 0.37832 | 0.29061 | 0.29525 | 0.22415 | 1.7098 | 0.86495 | 1.0701 | 0.38886 |
| 480 | 0.76054 | 1.7888 | 0.68375 | 0.67355 | 0.90714 | 0.64436 | 1.2456 | 0.64728 | 0.033978 |
| 481 | 0.27343 | 1.1683 | 1.8672 | 0.35558 | 0.55706 | 1.2397 | 0.010148 | 0.82804 | 1.8796 |
| 482 | 0.48131 | 1.7174 | 0.30035 | 1.2134 | 0.95634 | 0.23983 | 1.8621 | 1.4515 | 0.012037 |
| 483 | 0.47062 | 1.0471 | 1.4122 | 0.38699 | 0.70668 | 1.7682 | 0.23086 | 1.4377 | 0.075065 |
| 484 | 0.57273 | 0.33718 | 0.91157 | 1.0883 | 1.3818 | 0.39476 | 0.20505 | 0.46223 | 0.24808 |
| 485 | 0.75136 | 1.5607 | 0.94427 | 1.008 | 1.6033 | 1.9357 | 1.4512 | 0.30522 | 0.58997 |
| 486 | 0.34949 | 0.11038 | 1.6946 | 1.4952 | 1.1625 | 1.8397 | 1.5256 | 0.66828 | 0.14959 |
| 487 | 0.2682 | 0.42413 | 1.5372 | 0.64597 | 1.9473 | 0.82114 | 1.9405 | 1.2997 | 0.14713 |
| 488 | 0.33517 | 0.19352 | 1.553 | 1.3773 | 0.50339 | 1.0781 | 0.69367 | 1.4245 | 0.21593 |
| 489 | 0.70736 | 1.62 | 1.6856 | 0.9669 | 0.60223 | 1.9238 | 1.9194 | 1.0288 | 0.90905 |
| 490 | 0.31498 | 0.29761 | 1.1887 | 0.88141 | 0.10491 | 1.771 | 0.69887 | 1.5632 | 0.2353 |
| 491 | 0.69345 | 0.78141 | 0.70036 | 0.51633 | 0.52604 | 0.83067 | 1.4673 | 0.39097 | 0.23392 |
| 492 | 0.43645 | 0.55349 | 1.9103 | 0.94606 | 1.7288 | 0.39502 | 1.5242 | 1.1117 | 0.34289 |
| 493 | 0.31137 | 1.7201 | 0.078319 | 0.57204 | 1.8764 | 1.5571 | 0.97798 | 1.2904 | 0.007987 |
| 494 | 0.6459 | 1.252 | 1.3057 | 0.39474 | 1.4519 | 0.85149 | 0.92927 | 0.5418 | 0.28389 |
| 495 | 0.42329 | 0.2785 | 0.5447 | 1.4739 | 0.93722 | 0.52499 | 1.7919 | 1.1245 | 0.14463 |
| 496 | 0.73427 | 1.4969 | 1.3744 | 0.6916 | 1.1229 | 0.80187 | 0.98968 | 0.16912 | 0.20279 |
| 497 | 1.2607 | 1.2639 | 0.23834 | 0.94047 | 0.68403 | 1.8938 | 1.2619 | 0.042536 | 0.97106 |
| 498 | 0.62092 | 1.2956 | 0.45703 | 0.60824 | 0.82021 | 1.0712 | 1.0227 | 1.3571 | 0.27692 |
| 499 | 0.39933 | 0.62072 | 0.60628 | 0.79291 | 0.082177 | 1.4094 | 1.8111 | 1.2871 | 0.30096 |
| 500 | 1.1761 | 0.087331 | 0.65776 | 0.75333 | 0.029349 | 1.9435 | 1.0861 | 0.034478 | 1.4107 |
| 501 | 0.56887 | 0.16728 | 0.88383 | 0.47548 | 1.3247 | 1.3972 | 1.4263 | 0.77606 | 0.63962 |
| 502 | 0.34732 | 1.6985 | 0.37341 | 1.5678 | 0.75069 | 0.42975 | 1.0119 | 1.5976 | 0.076789 |
| 503 | 0.46334 | 0.45987 | 0.89941 | 1.2325 | 1.6655 | 0.85326 | 0.8883 | 1.7053 | 0.34946 |
| 504 | 0.48011 | 0.11389 | 0.33657 | 0.63984 | 1.9539 | 0.9093 | 0.59206 | 0.68916 | 0.76483 |
| 505 | 0.77751 | 1.5068 | 1.1253 | 0.37073 | 1.6753 | 1.9877 | 1.0686 | 0.10054 | 0.56286 |
| 506 | 0.39115 | 0.78387 | 1.0287 | 0.75621 | 0.97173 | 1.5446 | 1.8376 | 0.84284 | 0.1325 |
| 507 | 1.0352 | 0.58855 | 0.52021 | 1.216 | 0.10759 | 0.52275 | 0.61239 | 0.077473 | 0.84105 |
| 508 | 0.60612 | 1.5392 | 1.6002 | 0.97303 | 1.6555 | 0.29924 | 1.4876 | 0.23246 | 0.13615 |
| 509 | 0.69735 | 1.0926 | 0.010489 | 0.58826 | 1.962 | 0.90338 | 1.3317 | 0.36962 | 0.32637 |
| 510 | 0.83846 | 1.1024 | 0.48457 | 0.822 | 0.30373 | 0.45163 | 1.9623 | 0.1675 | 1.0028 |
| 511 | 0.49617 | 1.709 | 0.82661 | 1.7732 | 0.25814 | 0.60797 | 0.024035 | 0.20595 | 0.61856 |
| 512 | 0.61103 | 1.4019 | 1.0167 | 0.76027 | 0.30445 | 0.54809 | 0.41521 | 0.31748 | 0.71007 |
| 513 | 0.63622 | 1.6601 | 1.6658 | 1.1766 | 1.0659 | 0.72426 | 1.7883 | 1.2346 | 0.64119 |
| 514 | 0.27757 | 0.46355 | 0.59747 | 1.6728 | 0.44723 | 1.74 | 0.26905 | 1.5285 | 0.001375 |
| 515 | 0.79141 | 1.1942 | 1.1652 | 0.43419 | 0.77835 | 1.6488 | 1.9653 | 0.56874 | 0.049347 |
| 516 | 0.81535 | 0.39364 | 1.7239 | 1.1757 | 0.51412 | 1.7781 | 1.5996 | 0.1624 | 0.1909 |
| 517 | 0.53311 | 0.85355 | 1.0252 | 1.0625 | 1.0246 | 0.66382 | 1.1533 | 0.074261 | 0.81216 |
| 518 | 0.49789 | 1.3097 | 1.7334 | 0.76351 | 1.5396 | 0.76547 | 0.47623 | 1.2981 | 0.3659 |
| 519 | 0.63164 | 1.6762 | 0.55885 | 0.47831 | 0.91256 | 1.6944 | 1.4198 | 0.43192 | 0.63827 |
| 520 | 0.45807 | 1.0188 | 1.9656 | 0.30691 | 1.3062 | 0.71948 | 0.35397 | 0.9061 | 0.28643 |
| 521 | 0.70273 | 0.27093 | 1.2553 | 1.0461 | 0.69019 | 0.84514 | 0.19578 | 1.221 | 0.31966 |
| 522 | 0.69024 | 1.7137 | 1.1185 | 0.80097 | 1.2409 | 1.4497 | 1.9403 | 1.5835 | 0.055531 |
| 523 | 0.65882 | 0.21497 | 1.4105 | 1.0861 | 0.65417 | 0.61987 | 1.2019 | 1.5166 | 1.0087 |
| 524 | 0.40559 | 0.86746 | 1.3412 | 0.99803 | 0.47973 | 0.10975 | 0.56456 | 1.2964 | 0.34379 |
| 525 | 0.68991 | 0.49339 | 1.1313 | 0.82124 | 1.5327 | 1.4425 | 1.2277 | 1.1895 | 0.15676 |
| 526 | 0.49535 | 0.89796 | 1.7682 | 1.5855 | 0.16907 | 0.17452 | 1.3741 | 0.70687 | 0.5477 |
| 527 | 0.9654 | 1.9741 | 0.11905 | 1.2364 | 0.08057 | 0.17363 | 0.11159 | 0.049934 | 0.44756 |
| 528 | 0.62874 | 0.37738 | 0.52577 | 0.84153 | 0.63584 | 0.38221 | 0.60533 | 1.0388 | 0.67237 |
| 529 | 0.59267 | 0.49958 | 1.4845 | 1.4245 | 1.7853 | 0.44776 | 0.39382 | 1.7874 | 0.83142 |
| 530 | 0.41771 | 1.5108 | 0.37815 | 1.0127 | 0.8383 | 1.6104 | 0.095248 | 1.6722 | 0.43082 |
| 531 | 0.6215 | 0.95472 | 1.8091 | 0.4924 | 1.093 | 0.47418 | 0.031537 | 0.2527 | 1.2958 |
| 532 | 0.57952 | 0.066513 | 0.85512 | 1.096 | 0.4561 | 0.60888 | 1.827 | 1.5382 | 0.49123 |
| 533 | 0.52971 | 1.4198 | 0.15243 | 1.0683 | 0.71115 | 1.7507 | 1.1462 | 0.62419 | 0.94017 |
| 534 | 0.55962 | 0.19338 | 0.40044 | 0.4118 | 1.6309 | 1.3226 | 1.9221 | 0.40782 | 1.0589 |
| 535 | 0.68523 | 0.3368 | 1.7655 | 0.93993 | 1.756 | 0.12428 | 0.71229 | 0.35279 | 0.57953 |
| 536 | 0.3206 | 0.60491 | 1.0683 | 0.6821 | 1.5688 | 0.61883 | 1.3743 | 0.90979 | 0.13719 |
| 537 | 0.46876 | 1.4328 | 0.45369 | 0.83809 | 1.9448 | 0.65952 | 1.6729 | 0.23774 | 0.092657 |
| 538 | 0.81654 | 0.4228 | 0.91169 | 0.65685 | 0.53548 | 1.4834 | 0.082767 | 0.040974 | 1.6336 |
| 539 | 0.49633 | 0.30955 | 0.30424 | 0.15982 | 1.0838 | 1.7319 | 1.4469 | 1.9642 | 0.042892 |
| 540 | 0.92277 | 0.93357 | 1.9197 | 1.3983 | 0.57384 | 0.70103 | 0.95867 | 0.26588 | 1.3242 |
| 541 | 0.71374 | 1.7263 | 1.4881 | 0.98401 | 0.72363 | 0.55379 | 0.027476 | 0.32253 | 1.1095 |
| 542 | 0.35565 | 1.6878 | 0.83012 | 0.92547 | 1.4705 | 1.551 | 0.21289 | 1.838 | 0.17159 |
| 543 | 0.85361 | 1.5479 | 0.92783 | 0.72498 | 0.51174 | 1.3746 | 1.9092 | 0.12539 | 1.1331 |
| 544 | 0.56464 | 1.682 | 1.9393 | 0.80554 | 0.043034 | 1.0729 | 1.2124 | 1.2879 | 0.63673 |
| 545 | 0.33826 | 1.9697 | 1.2772 | 0.53253 | 1.5603 | 1.1322 | 0.55733 | 0.9481 | 0.084984 |
| 546 | 1.0805 | 1.8921 | 0.20375 | 1.0891 | 0.42276 | 0.34446 | 0.69479 | 0.035165 | 0.80958 |
| 547 | 0.82589 | 0.84196 | 0.31094 | 1.3731 | 1.3427 | 0.42999 | 0.017649 | 0.097458 | 1.4347 |
| 548 | 0.56075 | 1.3341 | 0.4375 | 0.50242 | 0.76699 | 1.3066 | 1.5702 | 1.5366 | 0.34508 |
| 549 | 0.79762 | 0.070266 | 1.7486 | 0.56335 | 1.5983 | 1.4233 | 1.3026 | 0.12179 | 0.89957 |
| 550 | 0.72411 | 0.52277 | 1.805 | 0.76275 | 0.92999 | 0.99391 | 1.3753 | 0.19316 | 0.51739 |
| 551 | 1.03 | 1.8371 | 0.81663 | 1.189 | 1.0962 | 0.85587 | 1.4488 | 0.11832 | 0.3236 |
| 552 | 0.60636 | 1.0123 | 0.31544 | 1.057 | 1.6874 | 1.6346 | 0.71825 | 0.77577 | 0.58829 |
| 553 | 0.58254 | 1.4897 | 1.0735 | 1.2765 | 1.5226 | 0.3846 | 0.1656 | 0.5204 | 1.4447 |
| 554 | 0.68048 | 0.63069 | 1.7709 | 0.53594 | 1.0018 | 0.98906 | 0.18233 | 0.31199 | 1.0282 |
| 555 | 0.53716 | 1.5206 | 1.7094 | 1.3564 | 0.12452 | 0.48573 | 0.17094 | 0.83464 | 0.83459 |
| 556 | 1.4192 | 0.47684 | 1.3488 | 0.84414 | 0.82362 | 1.5409 | 0.91184 | 0.041097 | 1.1666 |
| 557 | 0.91387 | 1.5304 | 1.4103 | 1.382 | 1.8862 | 0.4919 | 1.0259 | 0.20857 | 0.25224 |
| 558 | 0.53383 | 0.2759 | 1.9674 | 1.216 | 1.3328 | 0.8803 | 0.058309 | 0.54061 | 0.37363 |
| 559 | 0.78562 | 0.2945 | 0.89935 | 0.95168 | 0.29434 | 1.2795 | 0.42187 | 0.28064 | 1.1998 |
| 560 | 0.5591 | 0.62622 | 1.5756 | 1.183 | 1.9533 | 0.18477 | 1.3869 | 0.02621 | 0.57605 |
| 561 | 0.67855 | 1.3786 | 0.80677 | 0.28008 | 1.527 | 1.9993 | 0.85393 | 0.22533 | 0.91633 |
| 562 | 0.84454 | 1.391 | 1.8099 | 1.6117 | 0.65522 | 0.20163 | 0.74359 | 0.20328 | 0.52089 |
| 563 | 0.53121 | 1.7185 | 0.56468 | 1.1805 | 0.26202 | 0.59044 | 1.3255 | 1.8476 | 0.45202 |
| 564 | 0.64044 | 0.80754 | 1.7888 | 0.20904 | 1.7326 | 1.3588 | 0.88597 | 0.53335 | 0.53763 |
| 565 | 0.52435 | 1.0416 | 1.166 | 1.1623 | 1.1136 | 1.2897 | 1.6042 | 0.40294 | 1.0284 |
| 566 | 0.4685 | 1.9866 | 1.0212 | 1.0075 | 1.9855 | 1.3736 | 1.2221 | 0.84677 | 0.16121 |
| 567 | 0.49846 | 0.23593 | 0.24966 | 0.768 | 1.9943 | 0.43188 | 1.0441 | 1.1312 | 0.50137 |
| 568 | 1.1969 | 0.21795 | 0.82541 | 0.99829 | 1.7938 | 0.085836 | 1.4286 | 0.038664 | 0.15443 |

**3. Supplementary Figures**

**
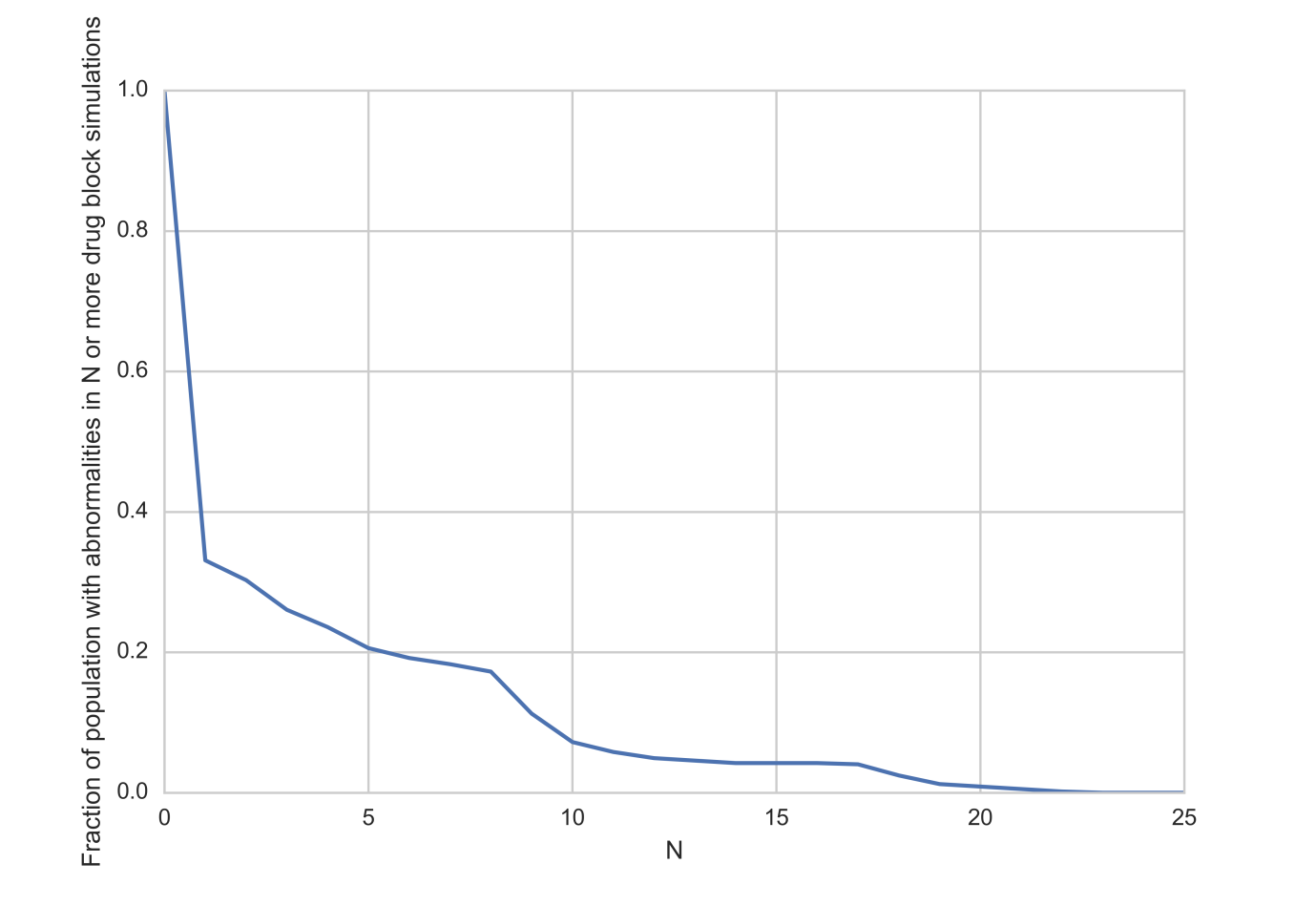
 Figure S1:** Fraction of population of models (n=568) that displayed abnormalities in N or more of the 96 drug block simulations performed. Particularly large drops in the number of models occur from N=0 to N=1 (threshold between NS and MS models) and from N=8 to N=10, which motivated setting threshold for HS models at N=10).

**
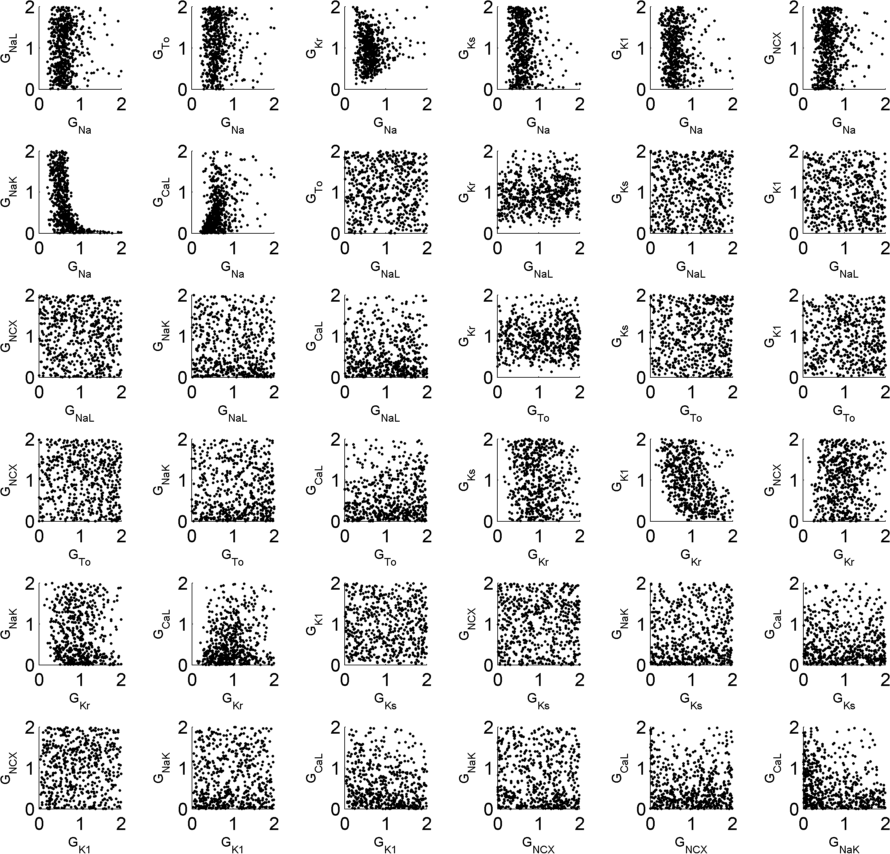
Figure S2**: Pairs of conductances across the population of models (n = 568). Plots show scaling values relative to the value of each conductance in the baseline ORd model. All possible pairs of conductances are shown.

**
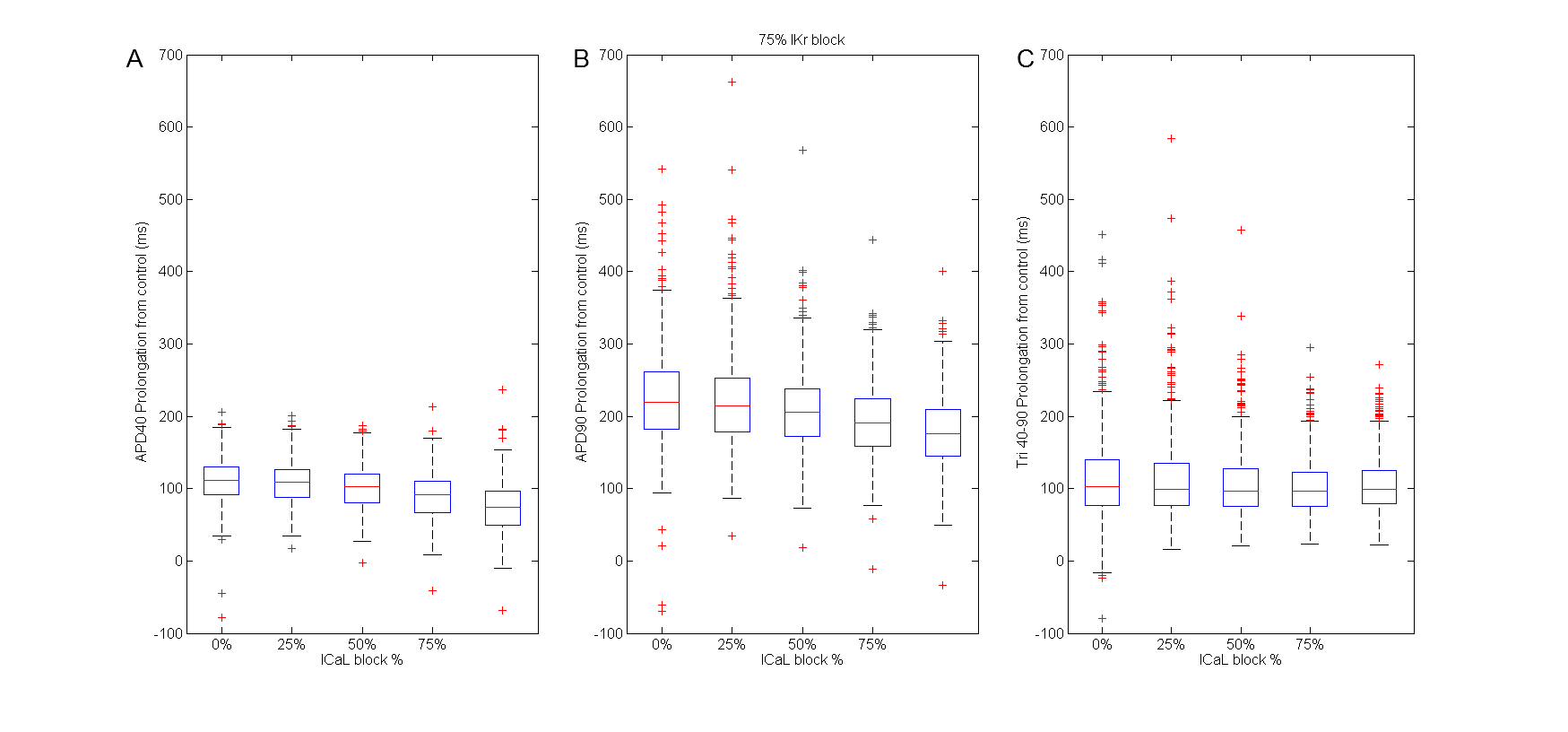
Figure S3:** Distributions of APD prolongation for **A:** APD_40_, **B:** APD_90_, and **C:** Triangulation_90-40_ observed across the population for different levels of I_CaL_ block during 75% I_Kr_ block. Models that displayed abnormalities were excluded, as repolarisation abnormalities can prevent calculation of a meaningful APD_90_ value if repolarisation does not complete before the next pacing cycle.

**Figure S4:** Magnitude of I_Na_, I_CaL_, I_NaK_, I_Kr_, I_Ks_, I_K1_ during repolarisation for NS and MS models in control conditions. Each current was averaged from the peak of the upstroke to APD_40_, and from APD_40_ to APD_90_, for each model.

**4. Supplementary References**

1. O'Hara T, Rudy Y. Quantitative comparison of cardiac ventricular myocyte electrophysiology and response to drugs in human and nonhuman species. Am J Physiol Heart Circ Physiol. 2012; 302(5):H1023-30.

2. Passini E, Mincholé A, Coppini R, Cerbai E, Rodriguez B, Severi S, Bueno-Orovio A. Mechanisms of pro-arrhythmic abnormalities in ventricular repolarisation and anti-arrhythmic therapies in human hypertrophic cardiomyopathy. J Moll Cell Cardiol. 2016; 96:72-81.

3. Livshitz L, Rudy Y. Uniqueness and stability of action potential models during rest, pacing, and conduction using problem-solving environment. Biophys J. 2009; 97(5):1265-1276.

4. Jost N, Virag L, Bitay M, Takacs J, Lengyel C, Biliczki P, et al. Restricting Excessive Cardiac Action Potential and QT Prolongation A Vital Role for IKs in Human Ventricular Muscle. Circulation. 2005; 112(10):1392-1399.

5. Pitt-Francis J, Pathmanathan P, Bernabeu MO, Bordas R, Cooper J, Fletcher AG, Mirams GR, Murray P, Osborne JM, Walter A, Chapman SJ, Garny A, van Leeuwen IMM, Maini PK, Rodríguez B, Waters SL, Whiteley JP, Byrne HM, Gavaghan DJ. Chaste: a test driven approach to software development for biological modelling. Comp Phys Comm. 2009;180:2452-2471.

6. Zhou X, Bueno-Orovio A, Orini M, Hanson B, Hayward M, Taggart P, Lambiase PD, Burrage K, Rodriguez B. In Vivo and In Silico Investigation Into Mechanisms of Frequency Dependence of Repolarization Alternans in Human Ventricular Cardiomyocytes. Circ Res. 2016; 118(2):266-78.

7. Bartos DC, Anderson JB, Bastiaenen R, Johnson JN, Gollob MH, Tester DJ, Burgess DE, Homfray T, Behr ER, Ackerman MJ, Guicheney P, Delisle BP. A KCNQ1 mutation causes a high penetrance for familial atrial fibrillation. J Cardiovasc Electrophysiol. 2013; 24(5):562-9.

8. Deo M, Ruan Y, Pandit SV, Shah K, Berenfeld O, Blaufox A, Cerrone M, Noujaim SF, Denegri M, Jalife J, Priori SG. KCNJ2 mutation in short QT syndrome 3 results in atrial fibrillation and ventricular proarrhythmia. Proc Natl Acad Sci U.S.A. 2013; 110(11):4291-6.

9. Hancox JC, Kharche S, El Harchi A, Stott J, Law P, Zhang H. In silico investigation of a KCNQ1 mutation associated with familial atrial fibrillation. J Electrocardiol. 2014 Mar-Apr; 47(2):158-65.

10. Harrell DT, Ashihara T, Ishikawa T, Tominaga I, Mazzanti A, Takahashi K, Oginosawa Y, Abe H, Maemura K, Sumitomo N, Uno K, Takano M, Priori SG, Makita N. Genotype-dependent differences in age of manifestation and arrhythmia complications in short QT syndrome. Int J Cardiol. 2015; 190:393-402.

11. Hasegawa K, Ohno S, Ashihara T, Itoh H, Ding WG, Toyoda F, Makiyama T, Aoki H, Nakamura Y, Delisle BP, Matsuura H. A novel KCNQ1 missense mutation identified in a patient with juvenile-onset atrial fibrillation causes constitutively open I Ks channels. Heart Rhythm. 2014; 11(1):67-75.

12. Jost N, Virág L, Comtois P, Ordög B, Szuts V, Seprényi G, Bitay M, Kohajda Z, Koncz I, Nagy N, Szél T, Magyar J, Kovács M, Puskás LG, Lengyel C, Wettwer E, Ravens U, Nánási PP, Papp JG, Varró A, Nattel S. Ionic mechanisms limiting cardiac repolarization reserve in humans compared to dogs. J Physiol (Lond.). 2013; 591(17):4189-206.

13. Marrus SB, Cuculich PS, Wang W, Nerbonne JM. Characterization of a novel, dominant negative KCNJ2 mutation associated with Andersen-Tawil syndrome. Channels (Austin). 2011; 5(6):500-9.

14. McBride CM, Smith AM, Smith JL, Reloj AR, Velasco EJ, Powell J, Elayi CS, Bartos DC, Burgess DE, Delisle BP. Mechanistic basis for type 2 long QT syndrome caused by KCNH2 mutations that disrupt conserved arginine residues in the voltage sensor. J Membr Biol. 2013; 246(5):355-64.

15. O'Hara T, Rudy Y. Arrhythmia formation in subclinical ("silent") long QT syndrome requires multiple insults: quantitative mechanistic study using the KCNQ1 mutation Q357R as example. Heart Rhythm. 2012; 9(2):275-82.

16. Romero L, Trenor B, Yang PC, Saiz J, Clancy CE. In silico screening of the impact of hERG channel kinetic abnormalities on channel block and susceptibility to acquired long QT syndrome. J Mol Cell Cardiol. 2014; 72:126-37.

17. Cummins MA, Dalal PJ, Bugana M, Severi S, Sobie EA. Comprehensive analyses of ventricular myocyte models identify targets exhibiting favorable rate dependence. PLoS Comput Biol. 2014; 10(3):e1003543.

18. Johannesen L, Vicente J, Gray RA, Galeotti L, Loring Z, Garnett CE, Florian J, Ugander M, Stockbridge N, Strauss DG. Improving the assessment of heart toxicity for all new drugs through translational regulatory science. Clin Pharmacol Ther. 2014; 95(5):501-8.

19. Lee HC, Rudy Y, Po-Yuan P, Sheu SH, Chang JG, Cui J. Modulation of KCNQ1 alternative splicing regulates cardiac IKs and action potential repolarization. Heart Rhythm. 2013; 10(8):1220-8.

20. Mirams GR, Davies MR, Brough SJ, Bridgland-Taylor MH, Cui Y, Gavaghan DJ, Abi-Gerges N. Prediction of Thorough QT study results using action potential simulations based on ion channel screens. J Pharmacol Toxicol Methods. 2014 Nov-Dec; 70(3):246-54.

21. Moreno C, de la Cruz A, Oliveras A, Kharche SR, Guizy M, Comes N, Starý T, Ronchi C, Rocchetti M, Baró I, Loussouarn G, Zaza A, Severi S, Felipe A, Valenzuela C. Marine n-3 PUFAs modulate IKs gating, channel expression, and location in membrane microdomains. Cardiovasc Res. 2015; 105(2):223-32.

22. Moreno JD, Yang PC, Bankston JR, Grandi E, Bers DM, Kass RS, Clancy CE. Ranolazine for congenital and acquired late INa-linked arrhythmias: in silico pharmacological screening. Circ Res. 2013; 113(7):e50-61.

23. Okada J, Yoshinaga T, Kurokawa J, Washio T, Furukawa T, Sawada K, Sugiura S, Hisada T. Screening system for drug-induced arrhythmogenic risk combining a patch clamp and heart simulator. Sci Adv. 2015; 1(4):e1400142.

24. Sadrieh A, Domanski L, Pitt-Francis J, Mann SA, Hodkinson EC, Ng CA, Perry MD, Taylor JA, Gavaghan D, Subbiah RN, Vandenberg JI, Hill AP. Multiscale cardiac modelling reveals the origins of notched T waves in long QT syndrome type 2. Nat Commun. 2014; 5:5069.

25. Trenor B, Gomis-Tena J, Cardona K, Romero L, Rajamani S, Belardinelli L, Giles WR, Saiz J. In silico assessment of drug safety in human heart applied to late sodium current blockers. Channels (Austin). 2013; 7(4):249-62.

26. Adeniran I, Hancox JC, Zhang H. Effect of cardiac ventricular mechanical contraction on the characteristics of the ECG: a simulation study. J Biomed Sci. 2013; 6(12):47.

27. Adeniran I, MacIver DH, Hancox JC, Zhang H. Abnormal calcium homeostasis in heart failure with preserved ejection fraction is related to both reduced contractile function and incomplete relaxation: an electromechanically detailed biophysical modeling study. Front Physiol. 2015; 6:78.

28. Walmsley J, Rodriguez JF, Mirams GR, Burrage K, Efimov IR, Rodriguez B. mRNA expression levels in failing human hearts predict cellular electrophysiological remodeling: a population-based simulation study. PLoS ONE. 2013; 8(2):e56359.

29. Elshrif MM, Shi P, Cherry EM. Electrophysiological properties under heart failure conditions in a human ventricular cell: A modeling study. The IEEE EMBC 2014. 2014; 4324-29.

30. Gomez JF, Cardona K, Romero L, Ferrero JM, Trenor B. Electrophysiological and structural remodeling in heart failure modulate arrhythmogenesis. 1D simulation study. PLoS ONE. 2014; 9(9):e106602.

31. Zhang J, Sacher F, Hoffmayer K, O'Hara T, Strom M, Cuculich P, Silva J, Cooper D, Faddis M, Hocini M, Haïssaguerre M, Scheinman M, Rudy Y. Cardiac electrophysiological substrate underlying the ECG phenotype and electrogram abnormalities in Brugada syndrome patients. Circulation. 2015; 131(22):1950-9.

32. Vicente J, Johannesen L, Galeotti L, Strauss DG. Mechanisms of sex and age differences in ventricular repolarization in humans. Am Heart J. 2014; 168(5):749-56.

33. Yang PC, Clancy CE. In silico Prediction of Sex-Based Differences in Human Susceptibility to Cardiac Ventricular Tachyarrhythmias. Front Physiol.2012; 3:360.

34. Christophe B. Simulation of early after-depolarisation in non-failing human ventricular myocytes: can this help cardiac safety pharmacology? *Pharmacol Rep*. 2013; 65(5):1281-93.

35. Pueyo E, Orini M, Rodríguez JF, Taggart P. Interactive effect of beta-adrenergic stimulation and mechanical stretch on low-frequency oscillations of ventricular action potential duration in humans. J. Mol Cell Cardiol. 2016; 97:93-105.
